# Subcellular view of host–microbiome nutrient exchange in sponges: insights into the ecological success of an early metazoan–microbe symbiosis

**DOI:** 10.1186/s40168-020-00984-w

**Published:** 2021-02-14

**Authors:** Meggie Hudspith, Laura Rix, Michelle Achlatis, Jeremy Bougoure, Paul Guagliardo, Peta L. Clode, Nicole S. Webster, Gerard Muyzer, Mathieu Pernice, Jasper M. de Goeij

**Affiliations:** 1 Department of Freshwater and Marine Ecology, Institute for Biodiversity and Ecosystem Dynamics, University of Amsterdam, Amsterdam, The Netherlands; 2 School of Biological Sciences, University of Queensland, Brisbane, Australia; 3 Centre for Microscopy, Characterisation and Analysis, The University of Western Australia, Perth, Australia; 4 The UWA Oceans Institute, The University of Western Australia, Perth, Australia; 5 The UWA School of Biological Sciences, The University of Western Australia, Perth, Australia; 6 Australian Institute of Marine Science, Townsville, Australia; 7 Australian Centre for Ecogenomics, University of Queensland, Brisbane, Australia; 8 Climate Change Cluster (C3), Faculty of Science, University of Technology, Sydney, Australia; 9 CARMABI Foundation, Piscaderabaai z/n, P.O. Box 2090, Willemstad, Curaçao

**Keywords:** Animal–microbe symbiosis, NanoSIMS, HMA–LMA, Dissolved organic matter (DOM), Particulate organic matter (POM), Nutrient translocation

## Abstract

**Background:**

Sponges are increasingly recognised as key ecosystem engineers in many aquatic habitats. They play an important role in nutrient cycling due to their unrivalled capacity for processing both dissolved and particulate organic matter (DOM and POM) and the exceptional metabolic repertoire of their diverse and abundant microbial communities. Functional studies determining the role of host and microbiome in organic nutrient uptake and exchange, however, are limited. Therefore, we coupled pulse-chase isotopic tracer techniques with nanoscale secondary ion mass spectrometry (NanoSIMS) to visualise the uptake and translocation of ^13^C- and ^15^N-labelled dissolved and particulate organic food at subcellular level in the high microbial abundance sponge *Plakortis angulospiculatus* and the low microbial abundance sponge *Halisarca caerulea.*

**Results:**

The two sponge species showed significant enrichment of DOM- and POM-derived ^13^C and ^15^N into their tissue over time. Microbial symbionts were actively involved in the assimilation of DOM, but host filtering cells (choanocytes) appeared to be the primary site of DOM and POM uptake in both sponge species overall, via pinocytosis and phagocytosis, respectively. Translocation of carbon and nitrogen from choanocytes to microbial symbionts occurred over time, irrespective of microbial abundance, reflecting recycling of host waste products by the microbiome.

**Conclusions:**

Here, we provide empirical evidence indicating that the prokaryotic communities of a high and a low microbial abundance sponge obtain nutritional benefits from their host-associated lifestyle. The metabolic interaction between the highly efficient filter-feeding host and its microbial symbionts likely provides a competitive advantage to the sponge holobiont in the oligotrophic environments in which they thrive, by retaining and recycling limiting nutrients. Sponges present a unique model to link nutritional symbiotic interactions to holobiont function, and, via cascading effects, ecosystem functioning, in one of the earliest metazoan–microbe symbioses.

Video abstract

**Supplementary Information:**

The online version contains supplementary material available at 10.1186/s40168-020-00984-w.

## Background

Sponges are increasingly recognised as key ecosystem engineers in many aquatic habitats, playing important roles in ecological processes, such as habitat provision and nutrient cycling [1, 2]. As filter-feeders *par excellence* [3, 4], the ecological success of sponges largely depends upon their ability to capture and transform a suite of organic and inorganic nutrients. Their unique and varied diet is related to the (inter)activity of the sponge host and its abundant and diverse microbial community, collectively termed the sponge holobiont. Sponges are considered one of the oldest extant metazoans, evolving more than 600 mya [5], and sponge–microbe associations are likely amongst the earliest of metazoan–microbe symbioses [6]. These symbioses are widely assumed to be mutualistic, but apart from a few notable exceptions [7–10], the nature of many of these beneficial interactions (mutualism, commensalism) has not been experimentally validated [6, 11].

The translocation or exchange of nutrients is a common feature of beneficial nutritional symbiosis [12]. The prevailing notion is that the sponge microbiome plays an important role in sponge health and nutrition [13] by extending the metabolic repertoire of the host [14, 15]. A wealth of -omics-based approaches have highlighted the metabolic potential of sponge symbionts, with putative benefits for the host ranging from chemical defence via the production of secondary metabolites [16, 17], supplying nutrition through the provision of fixed carbon or essential vitamins and amino acids [18, 19], to the recycling of host waste products [20, 21]. However, despite its identification as a priority research area, experimental evidence for many of these putative metabolic interactions is lacking [11]. The best characterised examples of beneficial sponge symbioses are between sponges and photoautotrophic symbionts, including cyanobacteria [22–24] and dinoflagellates (family *Symbiodiniaceae*) [7, 25]. Numerous studies have demonstrated the importance of these autotrophic symbionts in contributing to host nutrition, fitness, and growth through the translocation of photosynthetically fixed carbon and inorganic nitrogen [9, 10, 26, 27]. However, not all sponges contain abundant photoautotrophs and many rely predominantly on heterotrophic feeding on organic matter to meet their nutritional requirements. Furthermore, the benefits that microbial symbionts receive from their interaction with the host are less well-defined, but they are generally expected to profit from a nutrient-rich habitat within the sponge body. For example, in the deep-sea sponge *Geodia barretti*, microbially mediated nitrogen transformations were suggested to be fuelled by metabolic waste products of the host [28], but without direct evidence.

Sponges are opportunistic feeders capable of ingesting a wide spectrum of particulate organic matter (POM), but specialise in capturing cells < 10 μm, such as bacterio- and phytoplankton [13]. In the last few decades, it has become clear that dissolved organic matter (DOM) is also a major component of the diet (between 50 and 97 %) of many sponges, spanning tropical, shallow-water to cold, deep-sea species (reviewed in [2]). DOM represents the largest reservoir of organic carbon in the ocean [29] and is a relatively inaccessible food source for many other multicellular heterotrophic organisms. Furthermore, sponges convert DOM to POM—a more bioavailable food source for many organisms—via the so-called ‘sponge loop’ [30, 31], thereby aiding in the retention and recycling of nutrients in marine benthic ecosystems. Initially, it was assumed that DOM consumption by sponges was solely mediated by microbial symbionts, and therefore directly related to symbiont abundance [13, 32]. DOM-feeding was expected to be largely limited to high microbial abundance (HMA) sponges, who host symbiont densities 2–4 orders of magnitude higher than their low microbial abundance (LMA) counterparts [33–35]. However, compound-specific (e.g. stable isotope) tracer studies have shown that both sponge cells and associated microbes are involved in organic matter assimilation [36, 37], and recent nanoscale secondary ion mass spectrometry (NanoSIMS) studies have confirmed that both host choanocytes (sponge filtering cells) and symbionts can directly assimilate DOM [8, 38]. It has been hypothesised that microbially assimilated DOM will be translocated to the host [39], while host-processed POM will be recycled by microbial symbionts [13], but these metabolic interactions have not yet been tested.

**Fig. 1:**
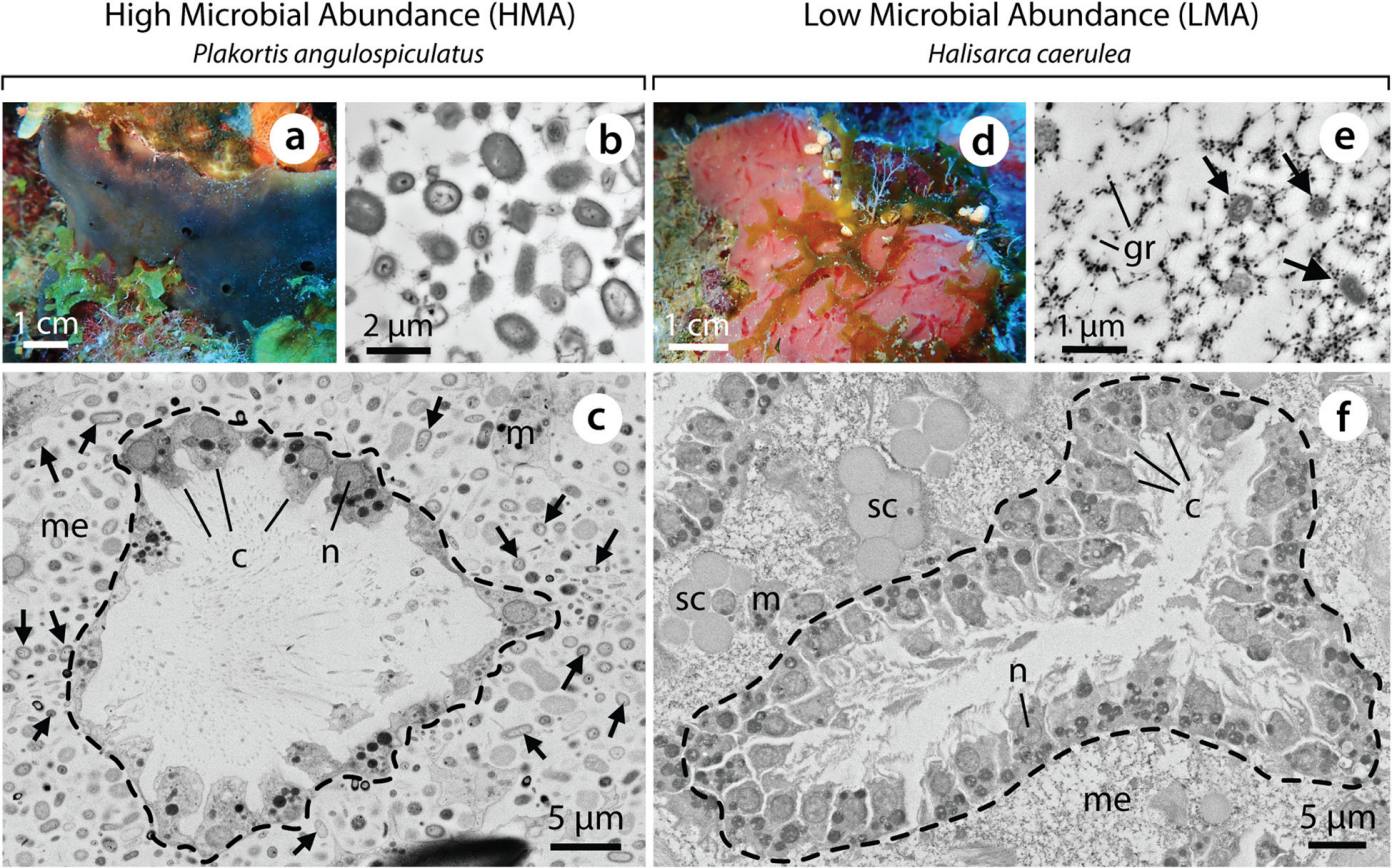
The encrusting tropical sponges *Plakortis angulospiculatus* (HMA, **a**–**c**) and *Halisarca caerulea* (LMA, **d**–**f**). Electron micrographs illustrating differences in the size and abundance of sponge-associated microbes (TEM, **b** and **e**), and density of choanocytes in choanocyte chambers (SEM, **c** and **f**), between the HMA and LMA species. c, choanocyte; gr, electron dense granule; m, mesohyl cell; me, mesohyl; n, nucleus; sc, spherulous cell. Dashed lines delineate choanocyte chambers and black arrows indicate sponge-associated microbes. *P. angulospiculatus* photograph kindly provided by Sara Campana

Here, we investigated the incorporation and processing of key heterotrophic dietary components by sponges at the holobiont and host cell and symbiont levels. We combined pulse-chase isotopic tracer techniques with NanoSIMS in order to trace the incorporation and fate of ^13^C- and ^15^N-labelled DOM and POM by the HMA sponge *Plakortis angulospiculatus* and the LMA sponge *Halisarca caerulea* (Fig. 1) over time. The use of NanoSIMS analysis allowed us to target the prokaryotic community and host cells of our sponges *in hospite* with subcellular resolution. The objectives of the study were to (i) examine the role of host cells and symbionts in organic matter incorporation and (ii) test for translocation between host and symbionts over time. This enabled us to disentangle complex interactions between host and microbiome in the uptake and exchange of heterotrophically acquired nutrients.

## Methods

### Sponge collection

This study was conducted at the Caribbean Research and Management of Biodiversity (CARMABI) field station, on the island of Curaçao (12°12′N, 68°56′W) between June and July 2018. The common Caribbean encrusting sponge species *Plakortis angulospiculatus* (HMA) and *Halisarca caerulea* (LMA) were collected from the fringing reefs in front of CARMABI and at station ‘Buoy 1’ (12°07′28.65′′N, 68°58′23.23′′W), located on the leeward side of Curaçao. These species were selected as they are good examples of both ends of the HMA–LMA spectrum (see Fig. 1), are easy to maintain and produce good histological sections. *Plakortis* is a cosmopolitan genus [40] and *P. angulospiculatus* is a widespread Caribbean species with available bacterial community data [41, 42]. For *H. caerulea*, an increasing body of information on physiology and ecology [30, 43], transcriptomics [44] and the microbial community [45], is available. Sponge individuals were collected by SCUBA diving at water depths of between 12 and 30 m and were removed from rock faces using a dive knife (*P. angulospiculatus*) or chiselled from dead coral plates and cleared of epibionts (*H. caerulea*) and cut into ~ 3–4 cm^3^ specimens with at least two functioning oscula (i.e. outflow opening; active pumping tested with fluorescein dye). Sponges were directly transferred to the wet-lab facilities of CARMABI and maintained in 100-L flow-through aquaria, supplied with water pumped from the adjacent reef at 10 m depth (flow rate approx. 3 L/min). Sponges were acclimatised for between 1 and 2 weeks and checked regularly to remove debris.

### Pulse-chase experiment with isotopically labelled tracers

To track the incorporation and fate of carbon (C) and nitrogen (N) over time, as important metabolic ‘currencies’ for DOM and POM nutrition [30, 31], a pulse-chase experiment was conducted using ^13^C- and ^15^N-labelled DOM and POM. Preparation of the isotopically labelled substrates is detailed in Additional file 1. Sponge individuals were incubated independently with DOM and POM over a 3-h pulse phase (0–3 h) and then transferred to flow-through aquaria with non-labelled water for the 45-h chase phase (3–48 h). Individuals were sampled at *t* = 15 min (*T*_0.25_), *t* = 30 min (*T*_0.5_), *t* = 1 h (*T*_1_), and *t* = 3 h (*T*_3_) during the pulse phase, and at *t* = 24 h (*T*_24_), and *t* = 48 h (*T*_48_) during the chase phase (*n* = 3 individuals per species, per food source, per time-point). A total of 84 sponges were used in the experiment, including control incubations with unlabelled DOM and POM (to determine background enrichment levels). Individual sponges were transferred to air-tight 2-L incubation chambers (see [30] for description of chambers) filled with 0.7-μm GF/F filtered seawater (FSW; 47 mm, Whatman). Chamber lids were equipped with a magnetic stirring device to ensure constant mixing and water flow during the incubations. Isotopically labelled DOM or POM was injected via syringe and the lid placed on each chamber ensuring no headspace. Food sources were added to give a final concentration of ~ 90 μM dissolved organic carbon (DOC) and ~ 1 × 10^6^ bacteria cells/mL in the chambers (see Additional file 1). An optical oxygen probe was inserted through an airtight port in the chamber lid and dissolved oxygen concentration (DO) was measured continuously (OXY-4 mini, PreSens) to ensure sufficient oxygenation and to monitor respiration throughout the incubations. Respiration rates were 48 ± 31 (mean ± SD) and 104 ± 59 μmol O_2_/g DW/h for the HMA sponge *P. angulospiculatus* and LMA sponge *H. caerulea*, respectively. Chambers were placed in a flow-through (3 L/min) aquarium to maintain ambient reef temperature and sponges were incubated in the dark during the pulse phase to exclude photosynthesis by photoautotrophs. At each sampling time-point, sponges were removed from the incubation chambers (pulse phase) or flow-through aquaria (chase phase) and rinsed in 0.7-μm FSW followed by Milli-Q water; chase sponges were first rinsed in label-free seawater before transfer from chambers to flow-through aquaria. Sponge planar surface area and thickness were then quantified from scaled photographs using ImageJ [46]. Three pieces of tissue per specimen (technical replicates) were removed using a biopsy punch (*P. angulospiculatus*, 2 mm ø, PFM Medical UK) or sterile scalpel blade (*H. caerulea*) and fixed in 2.5 % (v/v) glutaraldehyde + 1 % (w/v) paraformaldehyde in PHEM buffer (1.5× PHEM (60 mM PIPES, 25 mM HEPES, 10 mM EGTA, 2 mM MgSO_4_.7H_2_O), and 9 % (w/v) sucrose, pH 7.4) for electron microscopy and NanoSIMS analysis. The remaining tissue was transferred to pre-weighed cryovials using a sterile scalpel blade and stored at -20 °C for bulk C and N content and stable isotope analysis (for details see Additional file 1).

### Sample preparation for electron microscopy and NanoSIMS

Tissue samples were fixed for 12 h at 4 °C, rinsed three times with PHEM buffer (1.5× PHEM and 9 % (w/v) sucrose) and post fixed for 1.5 h with 1 % (w/v) osmium tetroxide in Milli-Q water. Samples were dehydrated in a graded series of ethanol and embedded in EPON Araldite. Embedded tissue was sectioned perpendicular to the surface of the sponge. Ultrathin (120 nm) and semithin (500 nm) sections were cut using a Reichert Ultracut S microtome. Ultrathin sections were transferred to finder grids (Electron Microscopy Sciences, Hatfield, PA, USA), stained with uranyl acetate and lead citrate, and imaged at 100 kV accelerating voltage using a Philips CM10 transmission electron microscope (TEM). These high-resolution images provided an initial characterisation of the tissue structure of both sponges, particularly regarding symbiont density and location (Fig. 1b, e). Semithin sections were transferred to silicon wafers, stained as above, and imaged with a Zeiss Sigma field emission scanning electron microscope (SEM) at 8 kV. Electron microscopy was performed at the Electron Microscopy Centre Amsterdam (EMCA). Regions of interest were identified by SEM and sample maps made to guide NanoSIMS analysis. One replicate per species and food source from incubations at *T*_0_, *T*_0.25_, *T*_0.5_, *T*_3_, and *T*_48_ was selected for NanoSIMS analysis.

### NanoSIMS analysis

To visualise the subcellular fate of incorporated ^13^C and ^15^N within sponge tissue, areas imaged by SEM were subsequently imaged with a NanoSIMS 50 ion probe (CAMECA, Paris, France) at the Centre for Microscopy, Characterisation and Analysis (University of Western Australia, Perth). Sections were gold coated (10 nm), then bombarded with a 16 keV Cs^+^ primary ion beam to detect the negative secondary ions ^12^C^12^C, ^13^C^12^C, ^12^C^14^N, ^12^C^15^N and ^31^P (for details, see Additional file 1). Between 5 and 8 different areas were scanned per sample. Images were processed using the OpenMIMS software plugin (National Resource for Imaging Mass Spectrometry, https://github.com/BWHCNI/OpenMIMS/wiki) for Fiji. Mass images were drift corrected, aligned and stacked, and presented as hue-saturation-intensity (HSI) images of the ^13^C/^12^C and ^15^N/^14^N ratios. Enrichment of ^13^C and ^15^N were quantified for the following regions of interest (ROI): (I) choanocytes, (II) all cells of the mesohyl (including archaeocytes and amoebocytes, but excluding spherulous cells which were present in *H. caerulea* only), and (III) sponge-associated microbes (Fig. 1). Additionally, areas of the mesohyl devoid of host cells (circles of < 1 μm diameter) and spherulous cells of *H. caerulea* were also included in the analysis and separately categorised, but will not be discussed in detail herein due to generally low enrichment throughout the pulse-chase experiment. All ROI were manually drawn over the NanoSIMS maps of ^12^C^14^N ions, using ^31^P and SEM images as a reference to identify cell types. The ROI selected for analysis captured the majority of enrichment in all areas of tissue scanned; only on few occasions were unidentifiable enriched cells excluded from analysis. A total of 14,979 ROI were analysed across 20 sponge individuals (Additional file 2: Table S1).

Unlabelled control sponge samples were analysed to obtain natural abundance ratios for each ROI category. Extracted isotopic ratios (*R*_sample_) for each ROI were multiplied by a correction factor, CF:

1 $$ \mathrm{CF}=\kern0.5em \frac{R_{\mathrm{sample}\kern0.5em \mathrm{IRMS}\kern0.5em \mathrm{yeast}}}{R_{\mathrm{sample}\kern0.5em \mathrm{yeast}}} $$

where *R*_sample IRMS yeast_ represents ratios obtained from bulk isotope ratio analysis of *Saccharomyces cerevisiae* (yeast), and *R*_sample yeast_ from ratios obtained from daily scans of yeast using NanoSIMS (see Additional file 1). Isotopic ratios were corrected and converted to ^13^C and ^15^N atom fractions (Atom%), *F*, which gives the amount of a specific atom (e.g. ^13^C) as a percentage of the total number of atoms (e.g., ^13^C + ^12^C):

2 $$ {F}_{\mathrm{sample}}=\left(\frac{R_{sample}}{R_{sample}+1}\right)\times \mathrm{CF}\kern2.5em \mathrm{Atom}\%=F\times 100 $$

A summary of ^13^C and ^15^N atom fractions and the number of ROI analysed are given in Additional file 2: Table S1. Individual ROI were deemed enriched in ^13^C and ^15^N if extracted Atom% values exceeded three times the standard deviation of mean isotopic values of corresponding ROI categories of unlabelled controls. The proportion of enriched versus non-enriched ROI populations was determined for choanocytes, mesohyl cells, and sponge-associated microbes (Additional file 2: Figure S1).

### Statistical analysis

Statistical analysis of the bulk tissue data was performed using SPSS (software V25). The effect of time on incorporation of DOM- or POM-derived ^13^C and ^15^N during the 3-h pulse phase was quantified using linear regression models for each species (Additional file 2: Table S2), and differences between regression coefficients were compared using univariate ANOVA in SPSS. Data met the assumptions of linearity, normality, homoscedasticity and sample independence.

NanoSIMS was foremost used here as an observational tool to visualise the uptake and transfer of organic ^13^C and ^15^N over time, and presentation of these results is limited to observed trends, since one sponge individual per time-point was analysed. However, as replication using NanoSIMS occurs at the single-cell level and not holobiont level (e.g. [7, 47, 48]) we present statistical analysis of the extracted single-cell data in Additional file 2: Tables S3 and S4.

## Results

### Bulk incorporation of organic matter by sponge holobionts

#### DOM

**Fig. 2:**
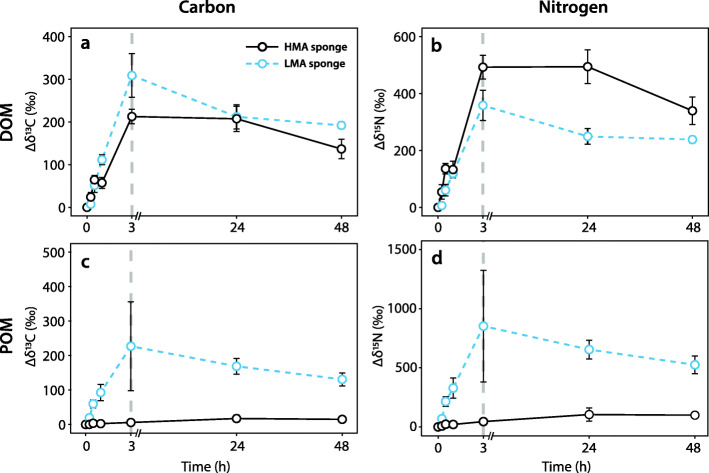
Stable isotope (^13^C and ^15^N) enrichment of sponge tissue during the 48-h pulse-chase experiment*.* Graphs show enrichment of DOM-derived ^13^C (**a**) and ^15^N (**b**), and POM-derived ^13^C (**c**) and ^15^N (**d**), into tissue of the HMA species *P. angulospiculatus* and the LMA species *H. caerulea.* Data display mean ± standard error (s.e.m) relative to unlabelled controls (Δδ^13^C and Δδ^15^N). The vertical dashed grey lines represent the end of the 3-h pulse phase, *n* = 3 per time-point, per species

The sponge species *P. angulospiculatus* (HMA) and *H. caerulea* (LMA) showed significant enrichment of DOM-derived ^13^C and ^15^N into their tissue over time (Fig. 2a, b; Additional file 2: Table S2). Stable isotope enrichment increased linearly during the 3-h pulse phase (regression analysis, all *p* < 0.001; Additional file 2: Table S2) in the sponge holobionts and gradually decreased during the subsequent chase phase when sponges were returned to label-free seawater (Fig. 2a, b). The rate of ^13^C incorporation during the pulse phase was significantly higher for the LMA species *H. caerulea* than for the HMA species *P. angulospiculatus* (ANOVA, *F* = 10.54, df = 1, *p* = 0.003) and the inverse relationship was true for ^15^N incorporation (ANOVA, *F* = 5.5, df = 1, *p* = 0.027).

#### POM

Both *P. angulospiculatus* and *H. caerulea* showed a linear increase of POM-derived ^13^C- and ^15^N-enrichment into their tissue during the 3-h pulse phase (regression analysis, all *p* < 0.001; Additional file 2: Table S2), but the HMA species *P. angulospiculatus* incorporated POM at a significantly lower rate than the LMA species *H. caerulea* for both ^13^C and ^15^N (ANOVA, *F* = 11.13 and *p* = 0.003, *F* = 11.34 and *p =* 0.002 for ^13^C and ^15^N, respectively) and enrichment was low across the pulse-chase experiment (Fig. 2c, d). *Halisarca caerulea* incorporated ^13^C and ^15^N in a pattern similar to that seen with DOM: a rapid increase during the 3-h pulse and gradual decrease during the subsequent chase phase (Fig. 2c, d).

### Single-cell analysis of organic matter incorporation

NanoSIMS analysis revealed substantial ^13^C- and ^15^N-enrichment derived from both isotopically labelled food sources into host cells and sponge-associated microbes of *P. angulospiculatus* and *H. caerulea*. Comparison of ^13^C/^12^C and ^15^N/^14^N ratio images with SEM micrographs enabled the patterns of enrichment to be ascribed to specific cells and subcellular structures within the sponge tissue (Fig. 1) and tracked through time.

#### DOM

**Fig. 3:**
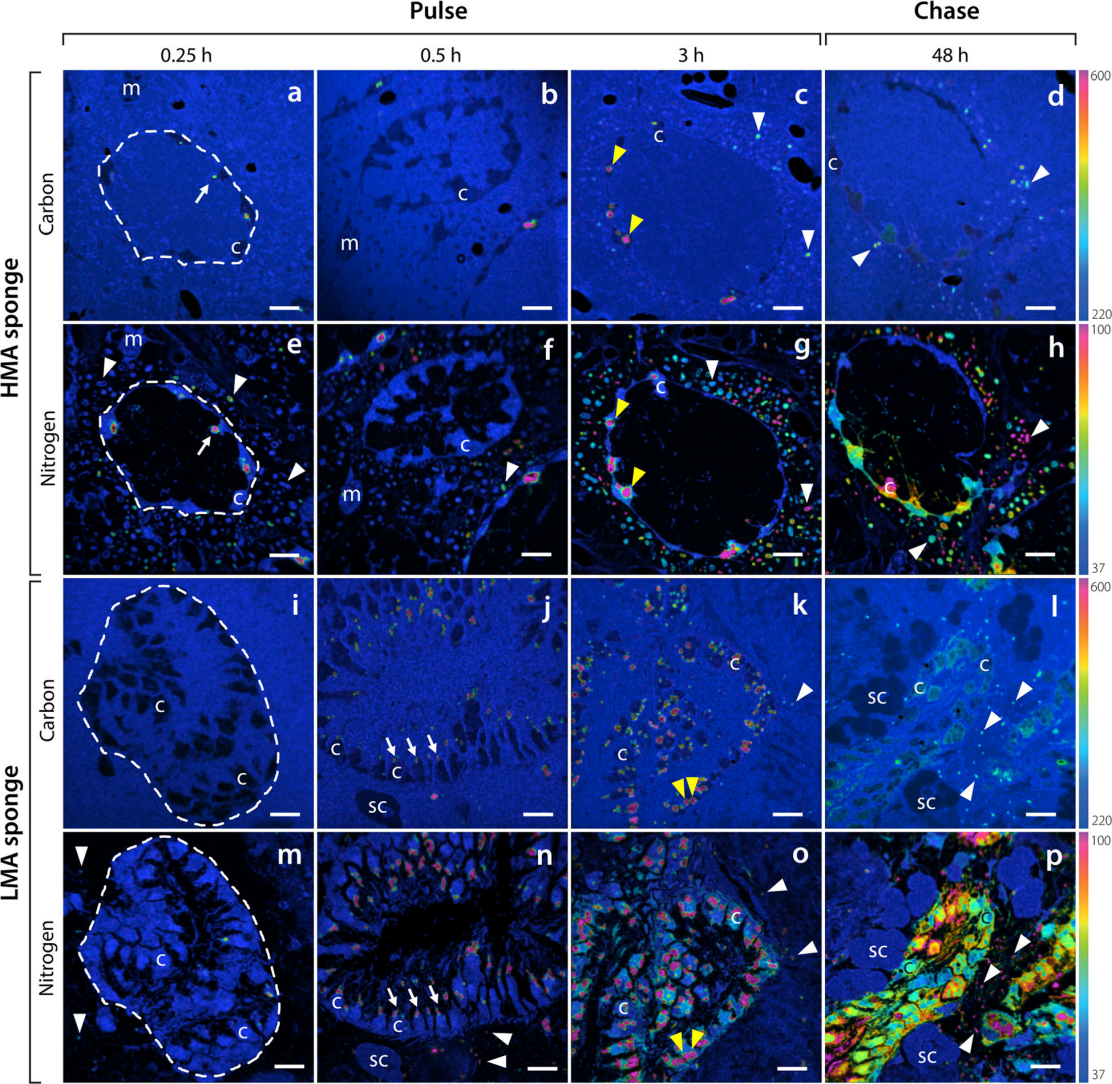
Uptake and distribution of ^13^C and ^15^N by host cells and sponge-associated microbes of the HMA species *P. angulospiculatus* (**a**–**h**) and LMA species *H. caerulea* (**i**–**p**) after a pulse of isotopically labelled DOM. NanoSIMS images show the distribution of ^13^C/^12^C (**a**–**d**, **i**–**l**) and ^15^N/^14^N (**e**–**h**, **m**–**p**) ratios after 0.25 h, 0.5 h, 3 h and 48 h. The colour scale represents enrichment relative to natural abundance ratios (in blue, 2 × 0.011 for ^13^C/^12^C and 0.0037 for ^15^N/^14^N). Incorporation of DOM can be traced into host choanocyte cells and sponge-associated microbes (white arrow heads) over time. Rapid uptake of DOM is evident in the apical tip of choanocytes after 0.25 and 0.5 h (white arrows). After 3 h, intracellular hotspots of ^13^C and ^15^N appear in choanocytes (yellow arrow heads), and during the chase period, the assimilated ^13^C and ^15^N is dispersed throughout these cells. c, choanocyte; m, mesohyl cell; sc, spherulous cell. Dashed lines delineate choanocyte chambers. Scale bars are 5 μm. For a summary of extracted values, see Additional file 2: Table S1

Within 15 min, sponge-associated microbes of both sponge species began to assimilate DOM-derived ^13^C and ^15^N. During this time, 19 and 51 % of the sponge-associated microbes of *P. angulospiculatus* and *H. caerulea* became enriched in ^15^N, respectively (Additional file 2: Figure S1). Nearly half of choanocyte cells (i.e. the sponge filtering cells; 43 and 51 % for ^13^C and ^15^N, respectively; Additional file 2: Figure S1) of the HMA species *P. angulospiculatus* became enriched during the first 15 min, with ‘hotspots’ of incorporation apparent in the cells (Fig. 3a, e), while incorporation of DOM into choanocytes of the LMA species *H. caerulea* was low (5 and 4 % of cells enriched for ^13^C and ^15^N, respectively; Additional file 2: Figure S1). After 30 min, numerous highly localised areas of ^13^C and ^15^N appeared in the apical tip of choanocyte cells of the LMA species (Fig. 3j, n), demonstrating direct incorporation of DOM. For both sponges, there was substantial enrichment of ^13^C and ^15^N into choanocytes and sponge-associated microbes at the end of the 3-h pulse, and choanocytes contained numerous large (1–2 μm) enrichment hotpots (Fig. 3). While some of these hotspots remained at the end of the chase phase, ^13^C and ^15^N became more homogenously distributed throughout choanocyte cells (Fig. 3).

**Fig. 4:**
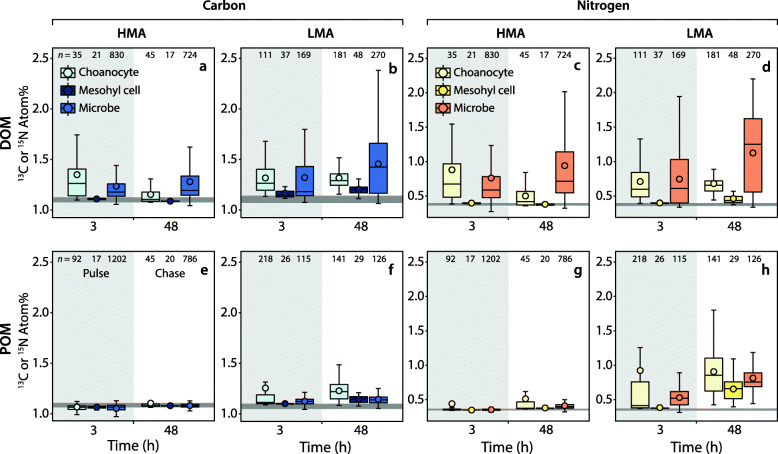
Quantification of ^13^C and ^15^N incorporation by host cells and sponge-associated microbes of the HMA species *P. angulospiculatus* and LMA species *H. caerulea* after a pulse of isotopically labelled DOM (**a**–**d**) and POM (**e**–**h**) using NanoSIMS. Shown are mean ^13^C- (**a**–**b**, **e**–**f**) and ^15^N- (**c**–**d**, **g**–**h**) enrichment values (in Atom%) for three regions of interest (ROI) within the sponge tissue: choanocyte, mesohyl cell and sponge-associated microbe, at the end of the pulse (shaded grey area) and chase period. Box plots display data as quartiles (lower and upper hinges represent the 25th and 75th percentiles) for each ROI and black circles represent mean values. The horizontal grey lines show the natural variation in ROI from unlabelled control sponges (as mean ± SD). For details see Additional file 2: Table S1. Note the different *y* axis scale between carbon and nitrogen values

The average ^13^C and ^15^N isotopic enrichment of choanocytes decreased during the chase phase in the HMA species *P. angulospiculatus*, while average enrichment in choanocytes of the LMA species *H. caerulea* remained the same for ^13^C and decreased slightly for ^15^N (Fig. 4a–d; Additional file 2: Table S1). A concurrent increase in the average isotopic enrichment of sponge-associated microbes occurred during the chase phase for both sponges, indicating translocation of C and N from choanocyte cells to sponge symbionts (Fig. 4a–d). Moreover, the percentage of sponge-associated microbes enriched in ^15^N increased from 86 to 95 % between 3 and 48 h in the HMA species, and from 58 to 88 % in the LMA species (Additional file 2: Figure S1). Mesohyl cells of the LMA species *H. caerulea* incorporated DOM-derived ^13^C and ^15^N during the 3-h pulse, and average enrichment further increased during the chase phase (Fig. 4b, d), indicating translocation of C and N from choanocytes to these cells. Enrichment of mesohyl cells in the HMA species *P. angulospiculatus* generally remained low throughout the pulse-chase (Additional file 2: Table S1).

#### POM

**Fig. 5:**
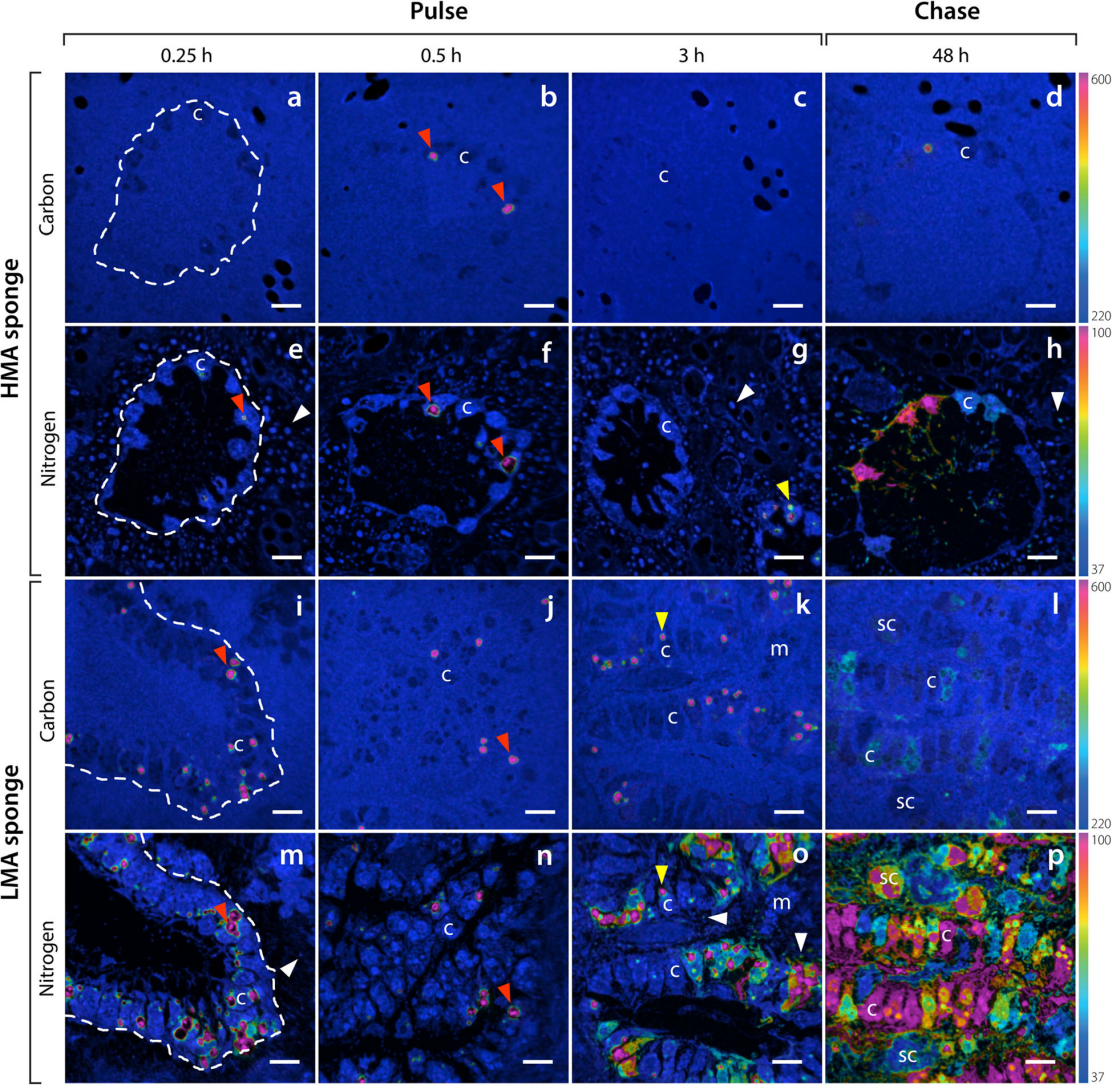
Uptake and distribution of ^13^C and ^15^N by host cells and sponge-associated microbes of the HMA species *P. angulospiculatus* (**a**–**h**) and LMA species *H. caerulea* (**i**–**p**) after a pulse of isotopically labelled POM (given as food bacteria). NanoSIMS images show the distribution of ^13^C/^12^C (**a**–**d**, **i**–**l**) and ^15^N/^14^N (**e**–**h**, **m**–**p**) ratios after 0.25 h, 0.5 h, 3 h and 48 h. The colour scale represents enrichment relative to natural abundance ratios (in blue, 2 × 0.011 for ^13^C/^12^C and 0.0037 for ^15^N/^14^N). Incorporation of POM can be traced into host choanocyte cells and sponge-associated microbes (white arrow heads) over time. POM is rapidly phagocytosed by choanocytes (red arrow heads). After 3 h, intracellular hotspots of ^13^C and ^15^N appear in choanocytes (yellow arrow heads) and sponge-associated microbes become enriched in ^13^C and ^15^N in the LMA sponge. During the chase period, assimilated ^15^N is dispersed throughout choanocyte cells. c, choanocyte; m, mesohyl cell; sc, spherulous cell. Dashed lines delineate choanocyte chambers. Scale bars are 5 μm. For a summary of extracted values, see Additional file 2: Table S1

As reflected in the bulk tissue data, incorporation of POM-derived ^13^C and ^15^N by the HMA species *P. angulospiculatus* was low across the pulse-chase (Fig. 5a–h). Sparse incorporation of labelled food bacteria can be seen in choanocytes of this HMA species at multiple time-points, with few individual food bacteria being phagocytosed (red arrows, Fig. 5b, e, f). Incorporated ^15^N became homogenously distributed throughout choanocytes at the end of the chase period (Fig. 5h). Average ^13^C- and ^15^N-enrichment of sponge-associated microbes remained low across the time series (Additional file 2: Table S1). The overall low incorporation rates of POM by *P. angulospiculatus* and variability between samples precludes reliable interpretation of potential translocation between cell types during the chase period.

**Fig. 6:**
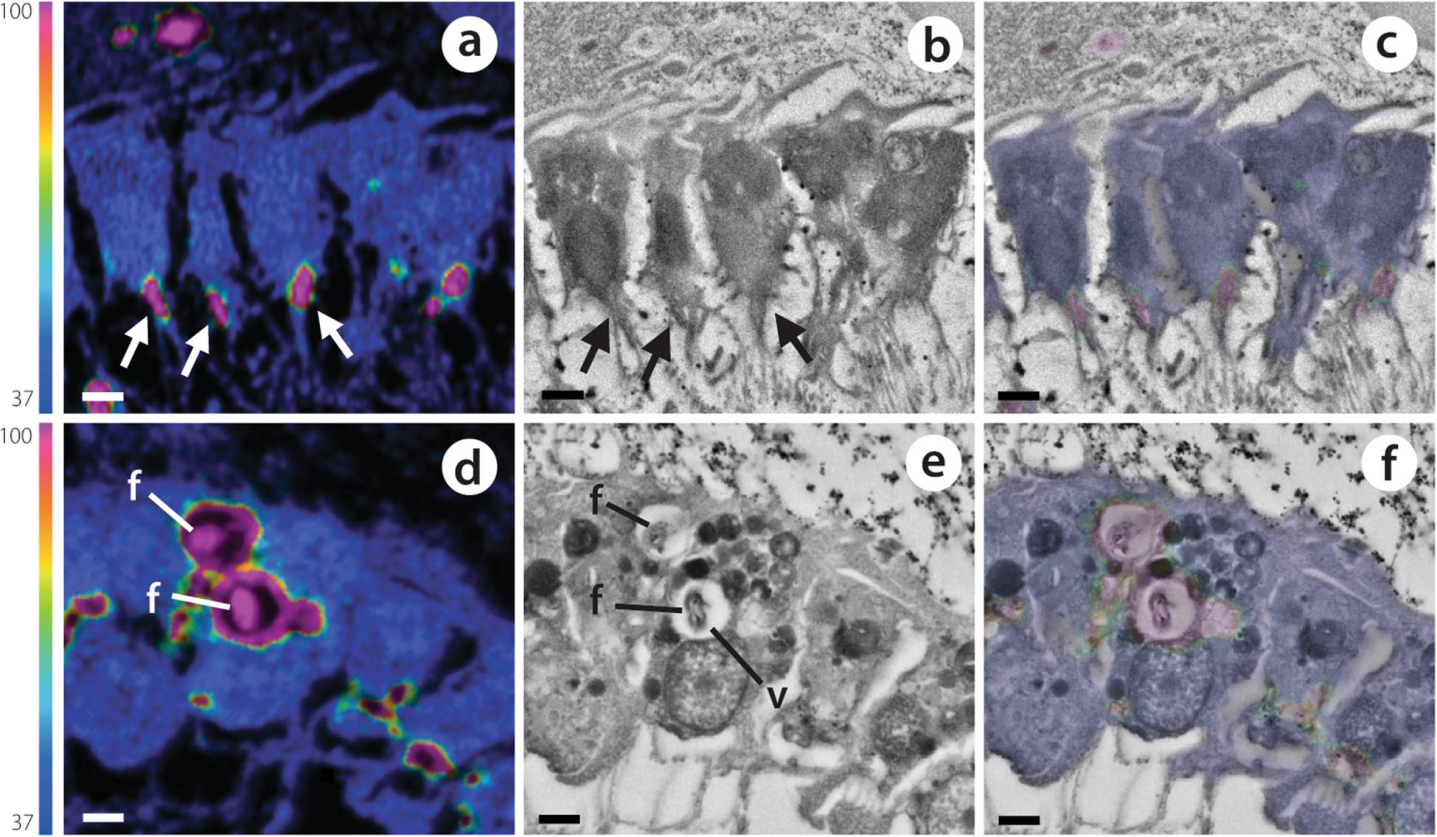
Contrasting mechanisms of isotopically labelled DOM (**a**–**c**) and POM (**d**–**f**) incorporation by choanocytes of the LMA species *H. caerulea*. Correlated NanoSIMS (**a**, **d**) and SEM images (**b**, **e**) with partial ^15^N-signal overlay (**c**, **f**) show the rapid internalisation of ^15^N labelled DOM and POM after 0.5 h and 0.25 h, respectively. DOM is incorporated into the apical tip of choanocytes (arrows), while food bacteria are phagocytosed by these filtering cells. The colour scale in **a** and **d** represents enrichment relative to natural abundance ratios (in blue, 0.0037). f, food bacteria; v, intracellular vesicle. Scale bars are 1 μm

A markedly different pattern was observed for the LMA species *H. caerulea*. Rapid incorporation of POM-derived ^13^C and ^15^N into choanocytes was evident, with enrichment in nearly all choanocytes within 15 min (100 and 90 % enrichment for ^13^C and ^15^N, respectively; Additional file 2: Figure S1). Individual labelled food bacteria were visibly phagocytosed by choanocytes during this time (Fig. 6d–f). Conversely, incorporation of POM-derived ^13^C and ^15^N into sponge-associated microbes was low during the early pulse incubations, indeed, after 30 min, ^13^C- and ^15^N-enrichment was detected in only 2 and 32 % of microbes, respectively (Additional file 2: Figure S1). At the end of the 3-h pulse, substantial incorporation of ^13^C and ^15^N was evident in choanocytes and sponge-associated microbes of the LMA species (Fig. 5k, o; Additional file 2: Table S1). Concentrated hotspots of enrichment were visible in choanocytes, but ^15^N had generally become dispersed throughout these cells (Fig. 5o). The presence of ^13^C and ^15^N in sponge-associated microbes after 3 h suggests translocation of metabolites from choanocyte cells, as symbionts are unable to phagocytose POM (see “Discussion” section). Both ^13^C and ^15^N became more uniformly distributed throughout the choanocytes during the chase period (Fig. 5l, p).

The average ^13^C and ^15^N enrichment of choanocytes in the LMA species *H. caerulea* decreased slightly during the chase period (Additional file 2: Table S1), although this trend was masked in Fig. 4 because enrichment at 3 h was highly variable, and included highly enriched choanocytes not captured in the boxplot distributions (Fig. 4f, h). The average isotopic enrichment of sponge-associated microbes increased during the chase period, and the proportion of enriched microbes also increased between 3 and 48 h, from 30 to 50 % for ^13^C, and 83 to 100 % for ^15^N (Additional file 2: Figure S1). Mesohyl cells of the LMA sponge became enriched in ^13^C and ^15^N during the 3-h pulse, and average enrichment further increased during the chase phase (Fig. 4f, h). The increased isotopic enrichment of sponge-associated microbes and cells of the mesohyl during the chase phase indicates translocation of C and N from choanocytes to these cells, since the majority of DOM incorporation was by choanocytes during the 3-h pulse (Fig. 5k, o) and their isotopic enrichment did not increase during the chase period.

## Discussion

Here, we show the uptake, processing, and transfer of dissolved and particulate food by sponge host and microbiome in one of the earliest metazoan–microbe symbioses. Consistent with recent findings [8, 38], both host cells and microbial symbionts of the HMA sponge *P. angulospiculatus* and the LMA sponge *H. caerulea* were actively involved in heterotrophic feeding processes. Contrary to the expectation that microbially assimilated DOM would be translocated to the host, we found experimental evidence for the translocation of both DOM- and POM-derived C and N from host cells to symbionts over time, demonstrating that the microbiome retains important nutrients within the holobiont via the recycling of host waste products. Our findings highlight the competitive strategy of the filter-feeding sponge holobiont, not only by tapping into a food resource (i.e. DOM) that is relatively inaccessible to many other marine heterotrophic metazoans, but by recycling potentially limiting nutrients via interactions with its microbial symbionts.

### The role of host and microbiome in organic matter uptake

#### DOM uptake

DOM was rapidly incorporated by the HMA species *P. angulospiculatus* and the LMA species *H. caerulea*, and bulk rates of incorporation into sponge tissue (1.1–1.5 μmol C_tracer_/mmol C_sponge_/h and 0.4–0.6 μmol N_tracer_/mmol N_sponge_/h; Additional file 2: Table S2) are in the range of rates presented for seven other tropical sponge species (0.3–1.2 μmol C_tracer_/mmol C_sponge_/h and 0.3–1.3 μmol N_tracer_/mmol N_sponge_/h) [30, 37], confirming the viability of our tested sponges. These findings contribute to a growing body of evidence that DOM-feeding by sponges is not related to the abundance of sponge-associated microbes [8, 38, 49, 50]. NanoSIMS analysis revealed that both symbionts and host choanocyte cells were actively involved in DOM uptake. The rapid ^13^C- and ^15^N-enrichment of symbiotic microbes (< 15 min) supports direct incorporation rather than transfer of nutrients by host cells. Indeed, incorporation of DOM by host cells was scarce during this initial time frame in the LMA species *H. caerulea* (Fig. 3i, m), whereas numerous enriched microbial cells were detected in regions distant from choanocyte chambers. Sponge-associated microbes may directly utilise limited amounts of DOM that enter the mesohyl through intercellular spaces between individual choanocytes or cells of the internal and external epithelia [51, 52]. Previous studies have revealed ‘gaps’ in the dermal membrane of sponges [53] and choanocytes have been shown to regulate their intercellular spaces to allow particle entry into the mesohyl [54]. Resident microbes likely incorporated low molecular weight (LMW, < 1 kDa) DOM, which is known to be rapidly (from min to h) assimilated [55, 56], as these labile molecules can readily diffuse across microbial membranes or be channelled via porins [57, 58].

The potential of choanocytes to directly incorporate DOM was first highlighted in *H. caerulea* after rapid incorporation of the cell proliferation marker BrdU—provided as a dissolved organic compound—into these cells [59]. Incorporation of DOM by choanocytes has since been confirmed in the bioeroding sponge *Cliona orientalis* [8] and the Mediterranean sponges *Aplysina aerophoba* and *Dysidea avara* [38] after labelling with a range of DOM sources. Here, our time-series approach allowed us to investigate DOM processing by choanocytes over time. We found hotspots of ^13^C and ^15^N incorporation in the apical tip of choanocyte cells of both sponges (particularly evident in *H. caerulea*, Fig. 6a–c) after 15 and 30 min of DOM-feeding. This strongly supports direct incorporation of DOM by choanocytes rather than the rapid translocation of metabolites from microbial symbionts located in the sponge mesohyl, as the apical surface of the choanocyte layer faces the surrounding seawater as it is drawn into choanocyte chambers. Likely, DOM is captured and retained within the apical collar complex of the choanocyte and subsequently endocytosed [8, 38]. Endocytosis of solutes can proceed via various pinocytotic pathways, including macropinocytosis or clathrin- or caveolae-mediated endocytosis [60]. The formation of membrane ‘ruffles’ found by Willenz and van de Vyver in 1982 [53], concurrent with the envelopment of latex beads by sponge exopinacocytes, may now be interpreted as evidence of macropinocytosis. Likewise, Laundon and colleagues [61] found macropinocytotic inclusions in choanocytes of a homoscleromorph sponge.

#### POM uptake

Our findings represent the first confirmation of host to symbiont translocation of POM-derived nutrients in a sponge, the LMA species *H. caerulea*, and provide support for the hypothesis that sponge symbionts gain nutritional benefit from their host-associated lifestyle. Uptake of POM in the LMA species *H. caerulea* was rapid (< 15 min; Fig. 2c, d), but NanoSIMS analysis showed that uptake was primarily confined to choanocyte cells, not microbial symbionts. Individual food bacteria were phagocytosed into large intracellular vesicles of choanocyte cells, in contrast to DOM incorporation into the apical collar via pinocytosis (Fig. 6). Substantial symbiont processing of POM did not occur until the end of the 3-h pulse, meaning that POM must first be captured, phagocytosed, and digested by choanocytes before intercellular microbes can scavenge C and N. This is consistent with current understanding that prokaryotes are incapable of phagocytosing bacteria [62]. POM-incorporation by choanocytes of the HMA species *P. angulospiculatus* was low throughout the pulse-chase experiment, and it may be that POM forms a minor part of the natural diet of this species. In subsequent natural diet experiments, we did observe that bacterial uptake rates for *P. angulospiculatus* are generally low (M. Hudspith and J.M. de Goeij, personal observation). However, we cannot exclude that *P. angulospiculatus* selected against our cultured POM source (*Vibrio caribbeanicus*), as sponges can selectively feed on particles, and different species may specialise in retaining specific portions of the planktonic community [54, 63, 64]. Therefore, we remain cautious in interpreting the ecological relevance of these findings, and our results should be compared with other planktonic sources.

### DOM and POM are differentially metabolised by the LMA sponge *Halisarca caerulea*

NanoSIMS images showed different spatial patterns of assimilation in choanocytes of the LMA sponge *H. caerulea,* indicating that the processing times of the two food sources differed in this species. Numerous intracellular hotspots (1–2 μm diameter) with substantial co-enrichment of C and N derived from DOM and POM could be seen within the choanocytes of both sponges after 3 h (yellow arrow heads, Figs. 3 and 5). These regions correlated with electron-dense vesicles in SEM images and represent putative endosomes or endolysosomes (DOM), and phagolysosomes (POM). However, in the POM-fed LMA species, these hotspots were in addition to a more uniform labelling of N throughout the choanocytes, unlike in DOM-fed sponges (Figs. 3o and 5o). This suggests that within 3 h, enzymatically degraded food bacteria had been digested into small molecules that had passed into the cytoplasm and entered biosynthesis pathways [65]. The comparatively rapid dispersal of N throughout choanocytes of the LMA species *H. caerulea* indicates that digestion and anabolic processing of particulate food was faster than that of dissolved food, and future studies should investigate if these two food sources differentially contribute to sponge nutrition.

NanoSIMS analysis indicated that DOM- and POM-derived C and N were translocated from choanocytes to mesohyl cells of *H. caerulea* during the chase period. This is consistent with the traditional view that digested material is passed from food capture cells to cells of the mesohyl (mainly amoebocytes) by transcytosis, whereby digestion is completed and metabolites distributed to other cell types [54]. No translocation to mesohyl cells was observed in the HMA species *P. angulospiculatus* within our experimental time frame, although variable and low uptake in the POM treatment may have masked potential transfer. In the LMA species *H. caerulea*, mesohyl cells, spherulous cells, and the mesohyl itself, showed a pronounced increase in POM-derived ^15^N enrichment between 3 and 48 h (Fig. 4h; Additional file 2: Table S1), indicating preferential utilisation of POM-derived N by cells of the mesohyl and the mesohyl matrix, which consists primarily of the polypeptides collagen, galectin, and fibronectin-like molecules [66]. Taken together, our results suggest that POM represents a high-quality food source for host anabolism (which may include predation on microbial symbionts [39, 67]), despite representing only a small portion of the natural organic matter pool and sponge diet [2, 50].

### The sponge microbiome as metabolic waste processors: commensalism or mutualism?

**Fig. 7:**
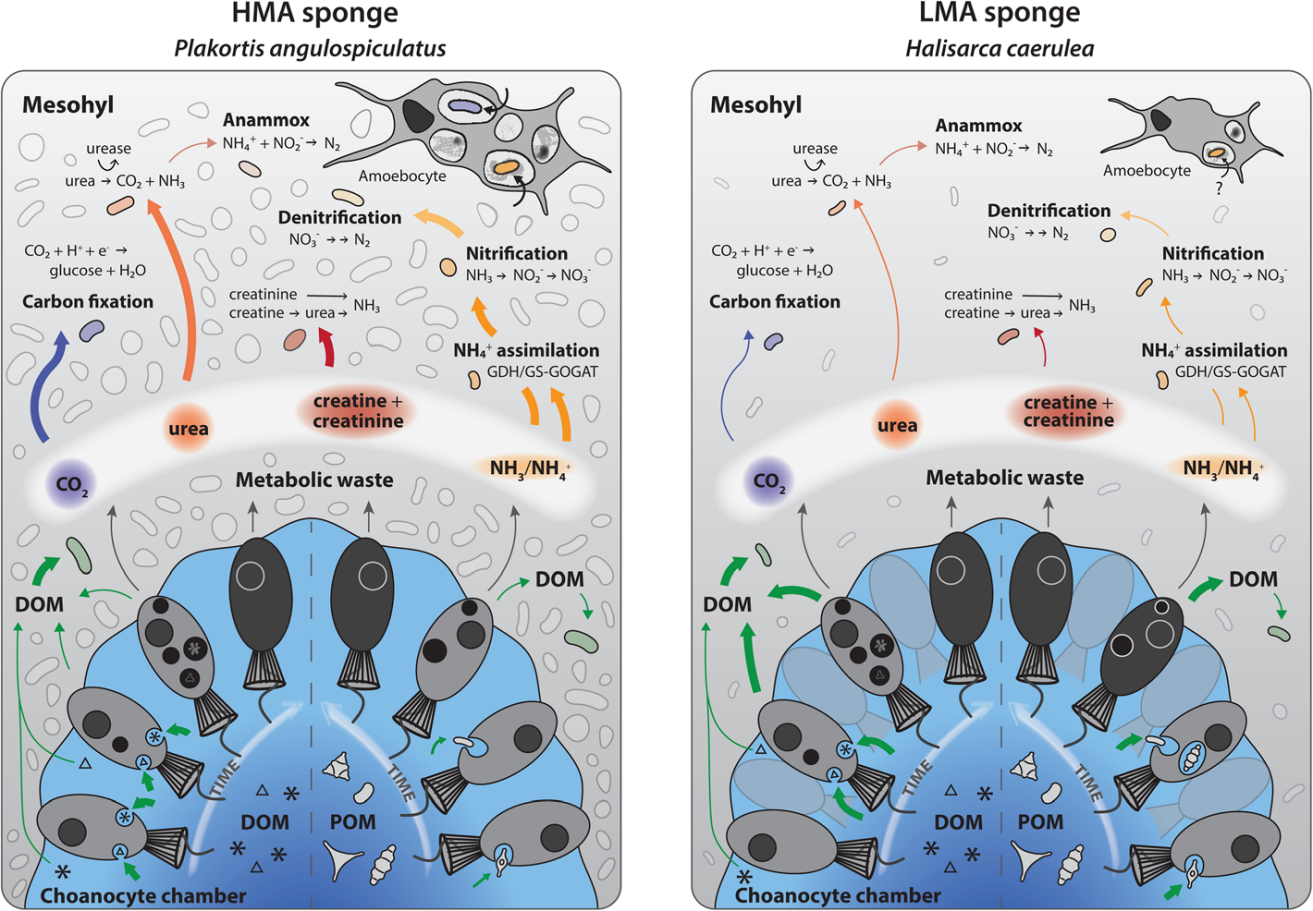
Schematic overview of organic matter uptake by the HMA species *P. angulospiculatus* and LMA species *H. caerulea* and hypothesised pathways of nutrient recycling by the microbiome. The width of arrows represents expected quantitative difference in fluxes: choanocyte processing pathways are based on NanoSIMS data (this study) and putative microbially mediated pathways are based on published data (partial transcriptional or genomic evidence included) [20, 28, 69–74]. Internal nitrogen recycling pathways are hypothesised to be elevated in HMA vs. LMA species [13, 49]. Host amoebocyte cells digest material passed from food capture cells (not shown), and in HMA species, these cells are prolific consumers of microbial symbionts (this study, [39])

Long-term associations between sponges and their symbiotic microbes are frequently described as mutualistic, although experimental evidence confirming that symbionts derive benefit from their host is scarce [7, 8]. Interactions have largely been addressed from the perspective of the host and the benefits received from symbiotic partners (e.g. photosynthates, vitamins, natural products [6]), whereas reciprocal benefits to symbionts are often presumed. It is generally hypothesised that symbiotic microbes profit from a high supply of nutrients and protection within the sponge mesohyl, but here we provide empirical evidence indicating that the prokaryotic communities of both *P. angulospiculatus* and *H. caerulea* directly benefit from the heterotrophic feeding activity of the host. NanoSIMS analysis indicated translocation of C and N from choanocytes to symbionts after DOM- and, restricted to the LMA species *H. caerulea*, POM-feeding (Fig. 4). This does not mean that translocation of metabolites from symbionts to the host did not occur, but rather was not observed under the current experimental design. Alongside the NanoSIMS observations, analysis of the single-cell data confirmed that the trends of increasing isotopic enrichment of microbial symbionts and mesohyl cells during the chase phase, indicative of translocation, were statistically significant (Additional file 2: Tables S3 and S4). However, biological replication is needed to robustly test the variation at the holobiont level. Our findings complement the wealth of genomic studies that have highlighted the potential of microbial symbionts to utilise host products (e.g. [68, 69]). Typical for LMA species, the microbiome of *H. caerulea* is largely dominated by *Proteobacteria* (*Alpha*- and *Gamma-*) [45] while *P. angulospiculatus* harbours a more diverse microbial community composed of *Chloroflexi, Acidobacteria*, and *Actinobacteria,* amongst other groups characteristic of HMA species [41]. These phyla comprise widespread and highly diverse lineages encompassing an extraordinary range of metabolic lifestyles. Thus, translocation of metabolites to symbiotic microbes may be fuelled by a multitude of different pathways, including the uptake of organic compounds released by host cells via incomplete oxidation [32] and the assimilation of host-derived metabolic waste (Fig. 7). Chemoautotrophic members of the sponge microbiome can utilise inorganic C produced during host respiration [70, 75, 76] and microbial N metabolism can be driven by the assimilation or remineralisation of sponge-excreted nitrogenous wastes [20, 71–73, 77]. Indeed, ammonia-oxidising archaea and bacteria have been detected in *H. caerulea* [74]. Given the complexity of the sponge holobiont, further studies are needed to link microbial identity to specific pathways of exchange and could employ RNA-based stable-isotope probing or correlative fluorescence in situ hybridisation and NanoSIMS. Emerging techniques such as time-of-flight (ToF) and hybrid SIMS [78] can also be utilised to determine the exact molecular nature of exchanged metabolites.

While our results show that symbionts of *P. angulospiculatus* and *H. caerulea* benefit from host feeding, we propose that the sponge host likely gains reciprocal benefit in a mutualistic manner. Utilisation of host waste products by the microbiome is advantageous for the host because toxic metabolites (e.g. ammonium) may be depurated and/or translocated back to host cells via microbial extracellular release or cell degradation [79]. No translocation of organic matter derived C and N from choanocytes to mesohyl cells was evident in the HMA species *P. angulospiculatus*, but we did observe amoebocyte-like cells of this species routinely phagocytosing multiple intercellular microbes (Additional file 2: Figure S2), demonstrating that symbiont–host transfer may also occur via phagocytosis or ‘farming’ of symbionts. This has been observed in deep-sea sponges [39, 67] and may provide an alternative source of nutrients for host cells in these oligotrophic environments. Additionally, it is plausible that the nutritional sponge host–microbiome interactions observed here are based on benefits received by the symbionts only (commensal) or that the relative benefits change according to intrinsic and external factors. Although further studies are needed to confirm if the internal recycling mechanisms observed here occur more broadly across other HMA and LMA species, we hypothesise that this is likely to be a general feature of sponge symbioses, given that the enrichment of genes related to the processing of host-derived compounds appears to be a core feature of sponge symbionts (e.g. [68, 80, 81]). The internal recycling of nutrients, mediated by mutualistic interactions with symbionts, would be advantageous for the sponge holobiont by limiting nutrient loss. Indeed, similar nutrient conserving mechanisms have been shown in other marine symbioses [79, 82]. Ultimately, these small-scale sponge–microbe interactions drive the internal cycling of the main biological elements (C, N and P) and are significant because they translate to whole sponge functioning and can therefore impact biogeochemical processes at ecosystem scales [83].

## Conclusions

Here, we visualise the role of sponge cells and microbial symbionts in the incorporation and exchange of two key dietary food sources: dissolved and particulate organic matter. Our results show that although microbial symbionts were involved in the uptake of DOM, sponge filtering cells appeared to be a major site of organic matter uptake in both the HMA sponge *P. angulospiculatus* and the LMA sponge *H. caerulea*, with translocation of C and N from host cells to microbial symbionts occurring over time. This indicates utilisation of host-derived waste products by the microbiome and provides evidence that symbionts benefit from their association with the sponge host. The interplay between the highly efficient filter-feeding host and its microbial symbionts—most notably by tapping into resources that others cannot readily utilise (i.e. DOM)—provides the sponge holobiont with a competitive edge over other heterotrophic marine organisms and enables them to persist and thrive in the many oligotrophic environments they are abundant in (e.g., the deep sea, the Mediterranean and coral reefs). Further studies quantifying the benefits or costs of symbiotic interactions will enhance our understanding of the influence of microbes on host ecology [25], and determining the response of these symbioses (and hence holobiont) to environmental perturbation will be crucial in the face of increasing anthropogenic pressures.

## Supplementary Information

**Additional file 1: Supplementary Materials and Methods.** A detailed description of methods used during (i) the preparation of isotopically labelled food substrates, (ii) NanoSIMS analysis, and (iii) bulk sponge tissue stable isotope analysis.

**Additional file 2: Supplementary Figures and Tables.** Supplementary figures (Figure S1 and Figure S2) and tables, including a summary of extracted data from NanoSIMS analysis (Table S1) and detailed statistical output (Table S2, S3, and S4).

## Data Availability

The datasets generated and analysed during the current study will be publicly available in the Dryad Digital Repository upon acceptance of the manuscript.

## Abbreviations

DOC: Dissolved organic carbon
DOM: Dissolved organic matter
DON: Dissolved organic nitrogen
HMA: High microbial abundance
LMA: Low microbial abundance
POC: Particulate organic carbon
POM: Particulate organic matter
PON: Particulate organic nitrogen
SEM: Scanning electron microscope
TEM: Transmission electron microscope
