# Supplementary material for: Subcellular view of host–microbiome nutrient exchange in sponges: insights into the ecological success of an early metazoan–microbe symbiosis
Source: Microbiome. 2021 Feb 14;9:44. doi: 10.1186/s40168-020-00984-w (PMC7883440; doi:10.1186/s40168-020-00984-w)
Supplement: Supplementary file 2 — Additional file 1: Supplementary Materials and Methods. A detailed description of methods used during (i) the preparation of isotopically labelled food substrates, (ii) NanoSIMS analysis, and (iii) bulk sponge tissue stable isotope analysis. [file 40168_2020_984_MOESM2_ESM.docx]

**Supplementary Materials and Methods**

*Preparation of isotopically labelled food substrates*

*DOM —* ^13^C- and ^15^N-labelled DOM was extracted from the cosmopolitan diatom *Phaeodactylum tricornutum*, as described by de Goeij et al. [1]. Briefly, batch cultures of *P. tricornutum* were grown on modified f/2 medium supplemented with 100 % NaH^13^CO_3_ and 50 % Na^15^NO_3_ (Cambridge Isotope Laboratories, 99 % ^13^C, 98 % ^15^N) and harvested in the late exponential phase. Cells were retained on a 0.2-µm cellulose nitrate filter (Sartorius), rinsed with artificial seawater (ASW), pelleted by centrifugation, and freeze-dried. Lyophilized cells were resuspended in Milli-Q water and sonicated, centrifuged, and the supernatant collected. The resulting pellet was subject to a further two rounds of extraction to maximize DOM production. The supernatant from each extraction was pooled and passed successively through a 0.7-µm GF/F filter (47 mm, Whatman) and 0.2-µm polycarbonate filter (47 mm, Whatman). The filtrate, representing the DOM, was collected and freeze-dried. Non-labelled DOM was produced as above using f/2 medium supplemented with 100 % NaH^12^CO_3_ and 100 % Na^14^NO_3_. A subsample of labelled and non-labelled DOM was taken for organic C and N content and isotopic composition analysis. The DOM was dissolved in Milli-Q, divided into 3 mL aliquots and stored at -20 °C until use in the pulse-chase experiment. DOM was fed to sponges at a final concentration of ⁓ 90 µM DOC (labelled DOM: 75 Atom% ^13^C, 99 Atom% ^15^N), which is within the range of ambient DOC concentrations previously measured in Curaçao (110 ± 18 μM DOC, mean ± SD) [2, 3].

*POM —* POM was supplied as isotopically enriched food bacteria. Cultures of ^13^C- and ^15^N-labelled bacteria were prepared using the Gram-negative bacterium *Vibrio caribbeanicus* (German Collection of Microorganisms and Cell Cultures DSMZ 23640). This marine non-pathogenic strain was chosen due to its relatively large size (1.0 µm wide and 2.5–3.1 µm long) [4], making it distinguishable in histological sections and NanoSIMS images. Bacteria were initially grown on TCBS agar for 48 h at 24 ºC and then transferred to Marine Broth (MB, Carl Roth, Germany). Approximately 0.5 mL of cell suspensions from exponentially growing MB cultures were transferred to 250 mL batches of M63 medium (pH 7.0, 13.6 g/L KH₂PO₄, 0.5 mg/L FeSO_4_.7H_2_O, 1 mM MgSO_4_) amended with 0.0001 % (w/v) thiamine and 1.5 % (w/v) NaCl. Non-labelled cultures for use in control incubations were grown on M63 media amended with glucose (0.2 % (w/v) final concentration) and NH_4_Cl (2.0 g/L), and labelled cultures were amended with ^13^C-glucose and ^15^NH_4_Cl (99 % ^13^C and 99 % ^15^N, respectively; Cambridge Stable Isotopes) at the same concentrations. Cultures were harvested in mid-exponential phase after 3–4 d incubation at 24 ºC. Cells were pelleted by centrifugation at 6000 x *g* for 10 min and washed twice by resuspension in 0.2-µm sterile-filtered seawater (SSW). The final pellets were resuspended in SSW, divided into 5 mL aliquots and stored at 4 ºC until use in the pulse-chase experiment. A subsample of labelled POM was taken for isotopic composition analysis. POM was fed to sponges at a concentration of ⁓ 1 x 10^6^ cells/mL (labelled POM: 92 Atom% ^13^C, 99 Atom% ^15^N); counts were made using epifluorescence microscopy and spectrophotometry. These concentrations were based on previously measured ambient seawater concentrations [3].

*NanoSIMS analysis*

Sections imaged with scanning electron microscopy (SEM) were subsequently analyzed using a NanoSIMS 50 ion probe (CAMECA, Paris, France). Selected analysis areas were identified using the CCD feature of the NanoSIMS 50 and then implanted to the same ion dose (6 x 10^16^ ions/cm^2^) prior to each acquisition. Images were typically acquired over a 40 x 40 µm area (512 x 512 pixels, 10 ms dwell time per pixel), with 8 layers scanned per image. For secondary ion imaging, the primary current was set to *c.* 2 pA using a 350 µm primary aperture, giving a spot size of *c.* 100 nm. The mass spectrometer was tuned to high mass resolution of *c.* 10000 (CAMECA definition), sufficient to separate all ion species of interest from any interferences, using an entrance slit of 30 µm and aperture slit of 200 µm. An internal yeast standard (*Saccharomyces cerevisiae*) was run every morning to monitor the drift of the machine and calibrate for instrument mass fractionation (IMF) [5] using the known isotopic composition of the yeast, which was based on isotope ratio mass-spectrometer (IRMS) data corrected to international standards (Vienna Pee Dee Belemnite and atmospheric nitrogen).

*Sponge holobiont stable isotope analysis*

To determine bulk enrichment of ^13^C- and ^15^N-labelled DOM and POM into the sponge holobiont, C and N content and isotopic ratios were measured using a Vario Isotope Tube Elemental Analyser (EA, Elementar GmbH, Germany) coupled with a BioVision IRMS (Elementar UK Ltd). Sponge tissue and samples of DOM and POM were freeze-dried, weighed, and homogenized. Subsamples for total N and δ^15^N analysis were directly weighed into tin boats, while subsamples for organic C (C_org_) content and δ^13^C values were first decalcified by acidification in 4 M HCl, diluted in Milli-Q water, and freeze-dried again prior to weighing into tin boats for EA-IRMS. Standard deviations of C and N content were < 4.8 % of measured concentrations and the precision of the IRMS was < 0.11 ‰ for repeated δ^13^C and δ^15^N measurements of the acetanilide standard. Stable isotope values were calculated and tracer incorporation quantified as described by de Goeij et al. [6]. Ratios were expressed as delta values (δ^13^C and δ^15^N) relative to Vienna Pee Dee Belemnite and atmospheric nitrogen. To calculate tracer incorporation rates, the linear regression equations describing the effect of time on δ^13^C and δ^15^N enrichment during the 3-h pulse phase (Additional file 2: Table S2) were used to calculate δ^13^C and δ^15^N enrichment values at 1 h per species, per food source. Briefly, delta values were converted to ^13^C and ^15^N atom fractions (F). The excess fractional abundance (E) was calculated, being the difference between the atom fraction of treatment and control sponges. Total ^13^C- or ^15^N-incorporation was calculated by multiplying E by the average C_org_ or N content of the HMA or LMA species, which was then divided by the labelling efficiency of the source (DOM or POM) to give total tracer incorporation. This was normalized to average C_org_ or N biomass of the HMA or LMA species, and expressed as μmol C or N_tracer_ per mmol C or N_sponge_ per hour.

**References**

1. de Goeij JM, van Oevelen D, Vermeij MJA, Osinga R, Middelburg JJ, de Goeij AFPM, et al. Surviving in a marine desert: The sponge loop retains resources within coral reefs. Science. 2013;342:108–10.

2. Mueller B, de Goeij JM, Vermeij MJ, Mulders Y, Van Der Ent E, Ribes M, et al. Natural diet of coral-excavating sponges consists mainly of dissolved organic carbon (DOC). PLoS One. 2014;9.

3. de Goeij JM, van den Berg H, van Oostveen MM, Epping EHG, van Duyl FC. Major bulk dissolved organic carbon (DOC) removal by encrusting coral reef cavity sponges. Mar Ecol Prog Ser. 2008;357:139–51.

4. Hoffmann M, Monday SR, Allard MW, Strain EA, Whittaker P, Naum M, et al. *Vibrio caribbeanicus* sp. nov., isolated from the marine sponge *Scleritoderma cyanea*. Int J Syst Evol Microbiol. 2012;62:1736–43.

5. Fitzsimons ICW, Harte B, Clark RM. SIMS stable isotope measurement: counting statistics and analytical precision. Mineral Mag. 2000;64:59–83.

6. de Goeij JM, Moodley L, Houtekamer M, Carballeira NM, van Duyl FC. Tracing 13C-enriched dissolved and particulate organic carbon in the bacteria-containing coral reef sponge *Halisarca caerulea*: Evidence for DOM-feeding. Limnol Oceanogr. 2008;53:1376–86.
