# Supplementary material for: Subcellular view of host–microbiome nutrient exchange in sponges: insights into the ecological success of an early metazoan–microbe symbiosis
Source: Microbiome. 2021 Feb 14;9:44. doi: 10.1186/s40168-020-00984-w (PMC7883440; doi:10.1186/s40168-020-00984-w)
Supplement: Supplementary file 3 — Additional file 2: Supplementary Figures and Tables. Supplementary figures (Figure S1 and Figure S2) and tables, including a summary of extracted data from NanoSIMS analysis (Table S1) and detailed statistical output (Table S2, S3, and S4). [file 40168_2020_984_MOESM3_ESM.docx]

**Supplementary Figures and Tables**


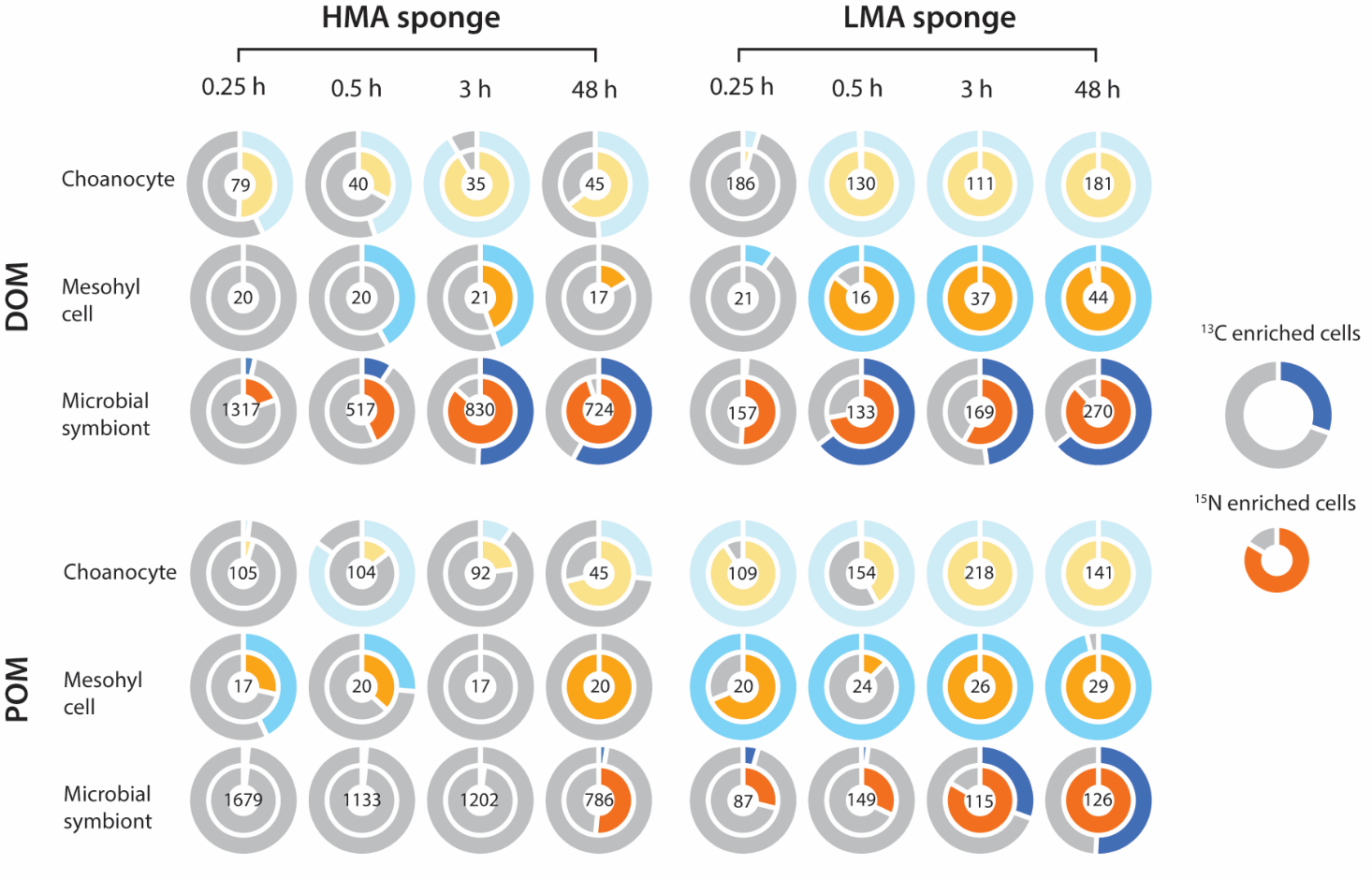


**Figure S1.** Percentage of ^13^C- and ^15^N-enriched host cells and microbial symbionts of the HMA species *P. angulospiculatus* and the LMA species *H. caerulea* after a pulse of isotopically labelled DOM and POM. Three regions of interest (ROI) within the sponge tissue (choanocyte, mesohyl cell, and microbial symbiont) were scanned using NanoSIMS. Mean enrichment of ^13^C and ^15^N (as Atom%) in each ROI was measured and shown are the distributions of enriched (colored) and non-enriched (grey) cells; individual ROI were considered enriched in ^13^C and ^15^N if extracted Atom% values were greater than three times the standard deviation of corresponding ROI of unlabelled controls. Shades of blue and orange are used to further distinguish ROI categories. The number of analyzed cells (*n*) per ROI category are shown in the centre of each pie chart.

**B**
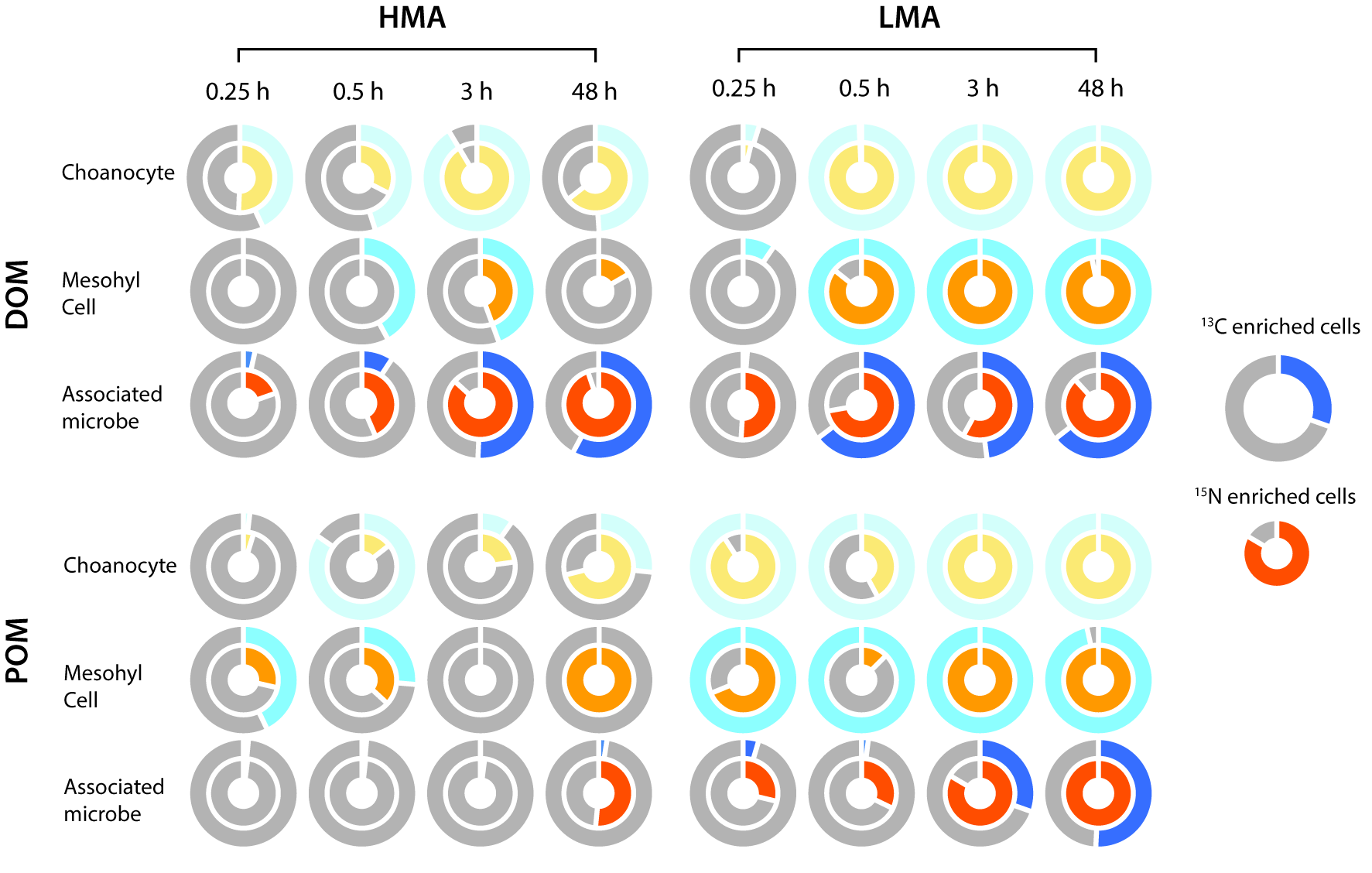


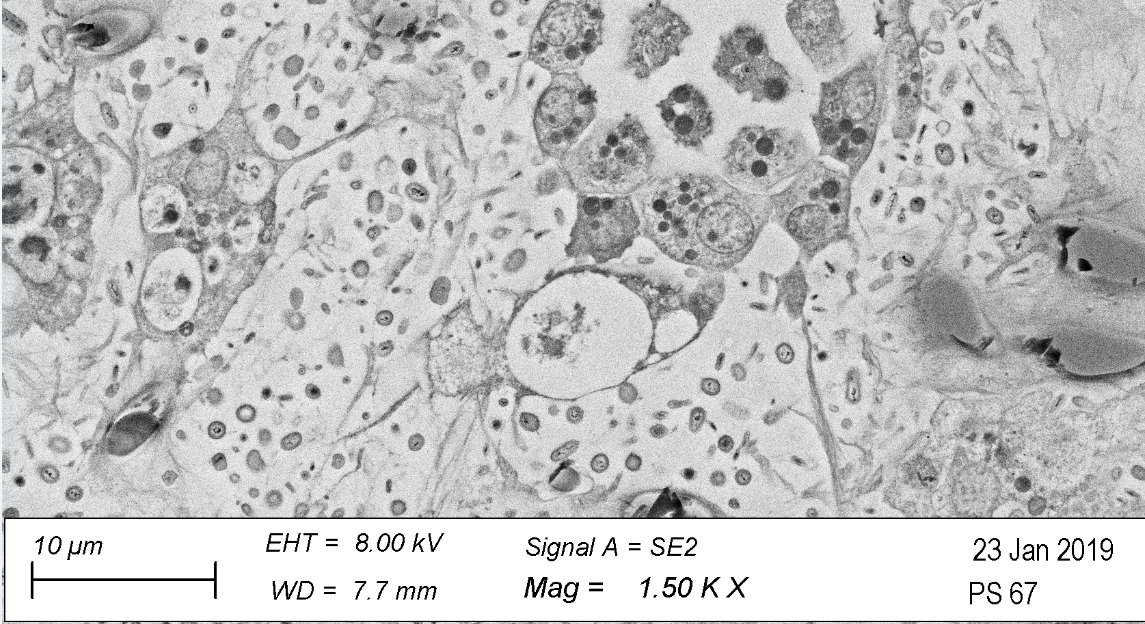

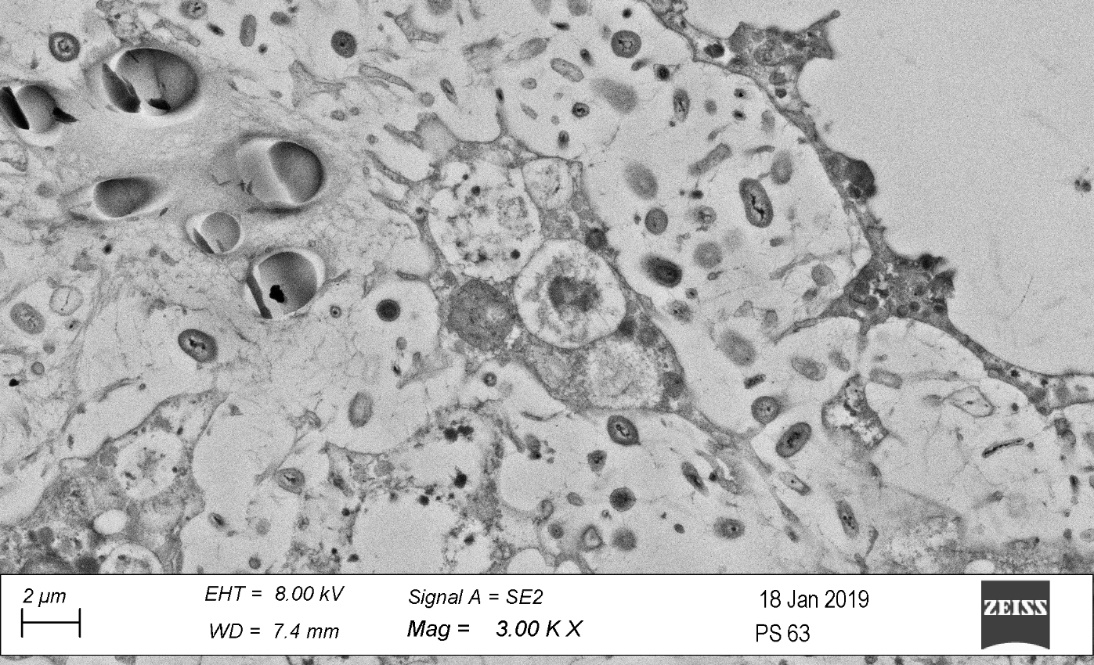


**em**

**h**

**h**

**em**

**v**

**v**

**2 μm**

**5 μm**

**a**
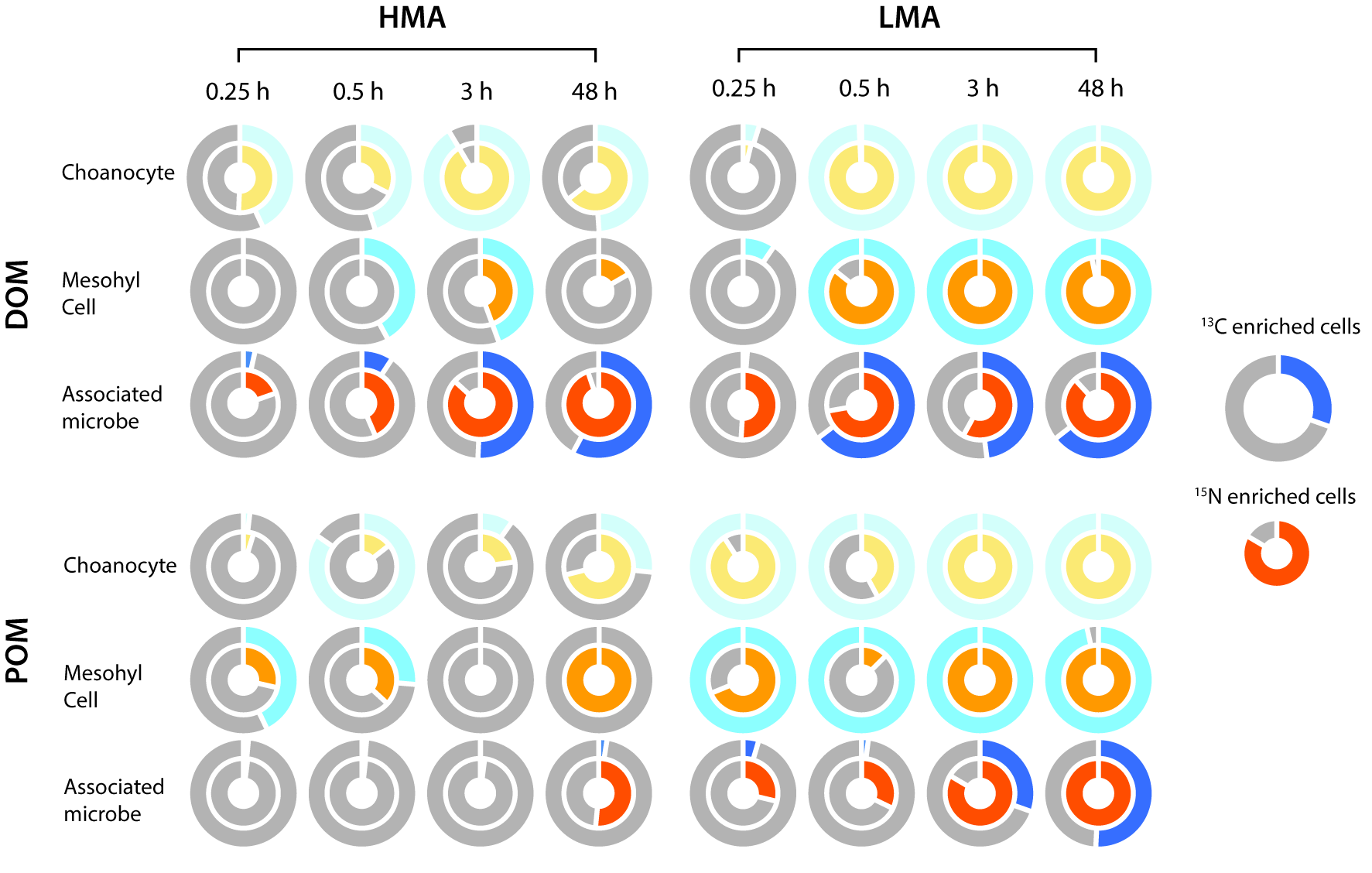


**b**
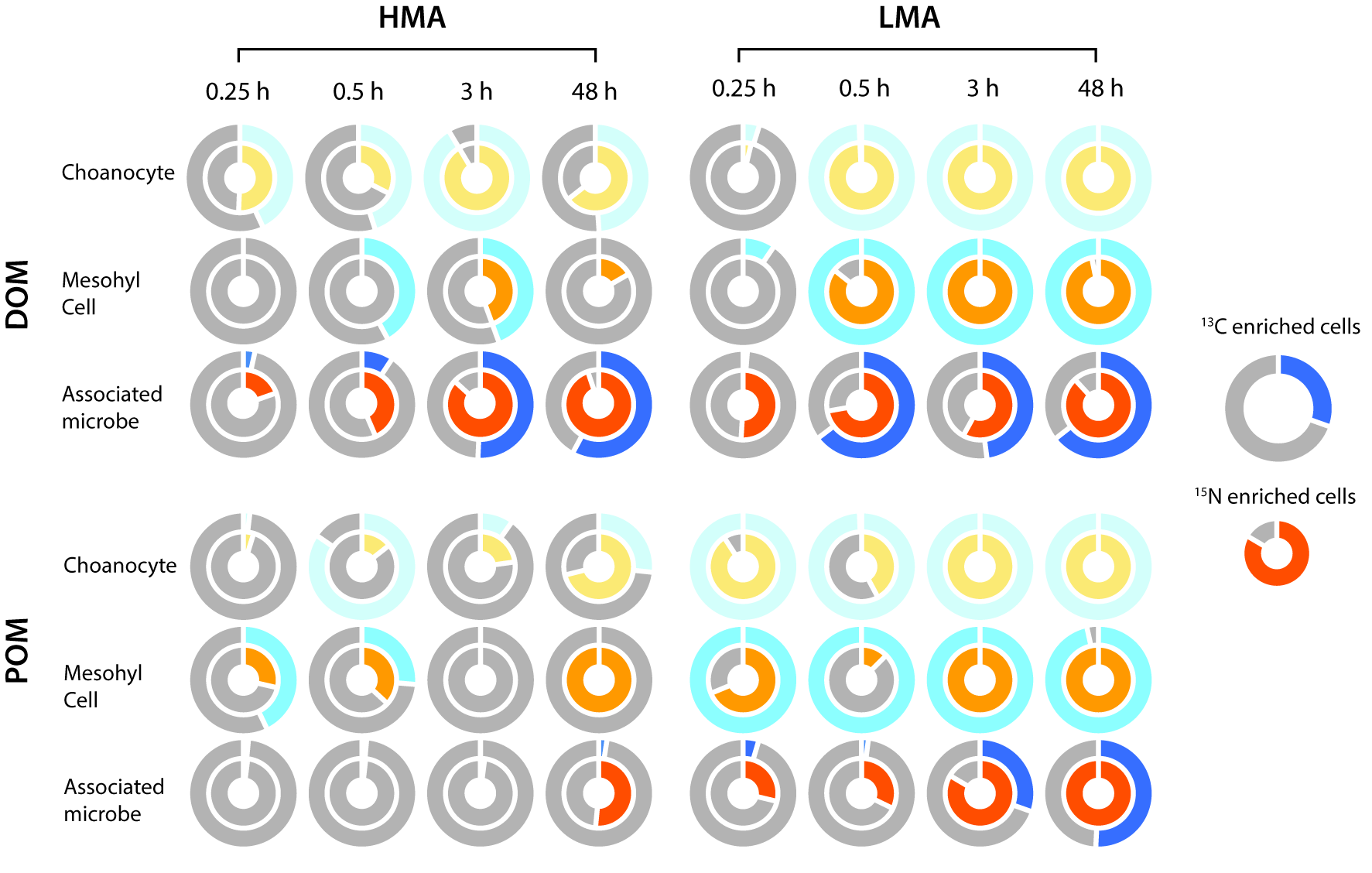


**Figure S2.** Phagocytosis o­f microbial symbionts by host cells of the HMA species *P. angulospiculatus*. SEM images (a, b) show host cells with multiple large intracellular vesicles containing engulfed symbiotic microbes in various stages of digestion. em, engulfed microbe; h, host cell; v, vesicle. Black arrows indicate symbiotic microbes in the mesohyl.

**Supplementary Table S1**

Summary of ^13^C- and ^15^N-atom fraction values (as Atom%) of each Region of Interest (ROI) in the sponges *Plakortis angulospiculatus* (HMA) and *Halisarca caerulea* (LMA) after incubation with isotopically labelled DOM or POM, as measured by NanoSIMS. *n* = number of analyzed cells of each ROI category.

|  |  |  |  | DOM | | | |  | POM | | | |
| --- | --- | --- | --- | --- | --- | --- | --- | --- | --- | --- | --- | --- |
|  |  |  |  | Atom% C | | Atom% N | |  | Atom% C | | Atom% N | |
|  | ROI | Time | *n* | Mean | SD | Mean | SD | *n* | Mean | SD | Mean | SD |
| HMA | Microbial | 0 | 1094 | 1.098 | 0.026 | 0.372 | 0.018 | 925 | 1.087 | 0.016 | 0.354 | 0.014 |
|  | Symbiont | 0.25 | 1317 | 1.111 | 0.027 | 0.408 | 0.083 | 1679 | 1.090 | 0.017 | 0.356 | 0.017 |
|  |  | 0.5 | 517 | 1.099 | 0.091 | 0.452 | 0.133 | 1133 | 1.100 | 0.015 | 0.359 | 0.019 |
|  |  | 3 | 830 | 1.234 | 0.169 | 0.761 | 0.513 | 1202 | 1.053 | 0.049 | 0.356 | 0.017 |
|  |  | 48 | 724 | 1.279 | 0.238 | 0.941 | 0.575 | 786 | 1.081 | 0.023 | 0.412 | 0.057 |
|  | Choanocyte | 0 | 73 | 1.093 | 0.006 | 0.372 | 0.005 | 57 | 1.075 | 0.006 | 0.356 | 0.005 |
|  |  | 0.25 | 79 | 1.171 | 0.199 | 0.512 | 0.327 | 105 | 1.082 | 0.005 | 0.358 | 0.006 |
|  |  | 0.5 | 40 | 1.135 | 0.121 | 0.462 | 0.246 | 104 | 1.111 | 0.098 | 0.430 | 0.508 |
|  |  | 3 | 35 | 1.349 | 0.400 | 0.880 | 0.813 | 92 | 1.066 | 0.117 | 0.441 | 0.367 |
|  |  | 48 | 45 | 1.153 | 0.100 | 0.501 | 0.181 | 45 | 1.105 | 0.063 | 0.513 | 0.289 |
|  | Mesohyl | 0 | 30 | 1.101 | 0.009 | 0.404 | 0.021 | 25 | 1.092 | 0.007 | 0.345 | 0.013 |
|  |  | 0.25 | 35 | 1.094 | 0.007 | 0.359 | 0.011 | 30 | 1.088 | 0.010 | 0.348 | 0.013 |
|  |  | 0.5 | 25 | 1.066 | 0.105 | 0.407 | 0.023 | 40 | 1.099 | 0.006 | 0.351 | 0.012 |
|  |  | 3 | 25 | 1.104 | 0.007 | 0.376 | 0.014 | 25 | 1.048 | 0.049 | 0.344 | 0.009 |
|  |  | 48 | 25 | 1.080 | 0.005 | 0.353 | 0.016 | 25 | 1.080 | 0.005 | 0.398 | 0.016 |
|  | Mesohyl | 0 | 20 | 1.097 | 0.005 | 0.374 | 0.008 | 17 | 1.080 | 0.007 | 0.352 | 0.002 |
|  | Cell | 0.25 | 20 | 1.093 | 0.007 | 0.36 | 0.001 | 17 | 1.087 | 0.010 | 0.355 | 0.002 |
|  |  | 0.5 | 20 | 1.077 | 0.069 | 0.379 | 0.003 | 20 | 1.099 | 0.002 | 0.355 | 0.002 |
|  |  | 3 | 21 | 1.108 | 0.007 | 0.396 | 0.013 | 17 | 1.070 | 0.015 | 0.349 | 0.002 |
|  |  | 48 | 17 | 1.086 | 0.008 | 0.376 | 0.022 | 20 | 1.081 | 0.005 | 0.379 | 0.012 |
| LMA | Microbial | 0 | 177 | 1.100 | 0.039 | 0.374 | 0.026 | 155 | 1.075 | 0.021 | 0.352 | 0.017 |
|  | Symbiont | 0.25 | 157 | 1.126 | 0.040 | 0.458 | 0.107 | 87 | 1.100 | 0.021 | 0.385 | 0.036 |
|  |  | 0.5 | 133 | 1.333 | 0.189 | 0.913 | 0.411 | 149 | 1.098 | 0.021 | 0.387 | 0.042 |
|  |  | 3 | 169 | 1.320 | 0.259 | 0.746 | 0.399 | 115 | 1.124 | 0.035 | 0.533 | 0.124 |
|  |  | 48 | 270 | 1.456 | 0.309 | 1.125 | 0.554 | 126 | 1.143 | 0.058 | 0.813 | 0.212 |
|  | Choanocyte | 0 | 64 | 1.093 | 0.005 | 0.372 | 0.002 | 130 | 1.066 | 0.005 | 0.356 | 0.002 |
|  |  | 0.25 | 186 | 1.094 | 0.019 | 0.371 | 0.010 | 109 | 1.182 | 0.202 | 0.714 | 0.857 |
|  |  | 0.5 | 130 | 1.287 | 0.352 | 0.557 | 0.371 | 154 | 1.130 | 0.149 | 0.502 | 0.656 |
|  |  | 3 | 111 | 1.316 | 0.176 | 0.710 | 0.322 | 218 | 1.254 | 0.369 | 0.924 | 1.294 |
|  |  | 48 | 181 | 1.316 | 0.124 | 0.680 | 0.186 | 141 | 1.227 | 0.103 | 0.907 | 0.350 |
|  | Mesohyl | 0 | 25 | 1.101 | 0.006 | 0.386 | 0.019 | 25 | 1.074 | 0.007 | 0.345 | 0.009 |
|  |  | 0.25 | 35 | 1.107 | 0.009 | 0.359 | 0.012 | 25 | 1.102 | 0.007 | 0.357 | 0.009 |
|  |  | 0.5 | 25 | 1.131 | 0.012 | 0.391 | 0.020 | 26 | 1.095 | 0.006 | 0.354 | 0.008 |
|  |  | 3 | 25 | 1.129 | 0.033 | 0.376 | 0.009 | 26 | 1.104 | 0.007 | 0.394 | 0.027 |
|  |  | 48 | 25 | 1.104 | 0.012 | 0.399 | 0.035 | 25 | 1.090 | 0.013 | 0.571 | 0.107 |
|  | Mesohyl | 0 | 41 | 1.092 | 0.005 | 0.371 | 0.003 | 20 | 1.065 | 0.005 | 0.355 | 0.002 |
|  | Cell | 0.25 | 21 | 1.094 | 0.012 | 0.370 | 0.003 | 20 | 1.095 | 0.005 | 0.364 | 0.005 |
|  |  | 0.5 | 16 | 1.149 | 0.052 | 0.405 | 0.071 | 24 | 1.095 | 0.004 | 0.360 | 0.005 |
|  |  | 3 | 37 | 1.165 | 0.052 | 0.399 | 0.010 | 26 | 1.101 | 0.010 | 0.385 | 0.036 |
|  |  | 48 | 44 | 1.204 | 0.053 | 0.462 | 0.082 | 29 | 1.145 | 0.045 | 0.655 | 0.167 |
|  | Spherulous | 0 | 9 | 1.094 | 0.003 | 0.369 | 0.002 | 16 | 1.069 | 0.005 | 0.352 | 0.001 |
|  | Cell | 0.25 | 12 | 1.096 | 0.006 | 0.367 | 0.001 | 3 | 1.094 | 0.001 | 0.359 | 0.001 |
|  |  | 0.5 | 6 | 1.123 | 0.012 | 0.375 | 0.008 | 10 | 1.092 | 0.004 | 0.358 | 0.001 |
|  |  | 3 | 24 | 1.115 | 0.005 | 0.385 | 0.003 | 4 | 1.098 | 0.003 | 0.373 | 0.002 |
|  |  | 48 | 16 | 1.111 | 0.029 | 0.376 | 0.032 | 21 | 1.154 | 0.067 | 0.605 | 0.184 |

**Supplementary Table S2**

Results of linear regression analysis modelling the effect of time on DOM- or POM-derived δ^13^C and δ^15^N enrichment by the sponges *Plakortis angulospiculatus* (HMA) and *Halisarca caerulea* (LMA) during the 3-h pulse phase (*n* = 15 per species, per food source). Models were used to calculate isotopic enrichment after 1 h, which were converted into tracer incorporation rates, expressed as μmol C or N_tracer_/mmol C or N_sponge_/h (details in Additional file 1). Adj R^2^ = adjusted R^2^_,_ CI = 95 % confidence intervals, Int = intercept. Values in bold are statistically significant (*p* < 0.05).

|  |  | Species | Slope | Int | t | CI (lower) | CI (upper) | Adj R^2^ | *p* value | Incorporation rate |
| --- | --- | --- | --- | --- | --- | --- | --- | --- | --- | --- |
|  |  |  |  |  |  |  |  |  |  |  |
| DOM | δ^13^C | HMA | 67.77 | -11.76 | 12.303 | 55.87 | 79.67 | 0.915 | **<0.0001** | 1.1 ± 0.2 |
|  |  | LMA | 104.35 | -22.23 | 10.616 | 83.11 | 125.58 | 0.889 | **<0.0001** | 1.5 ± 0.3 |
|  | δ^15^N | HMA | 158.12 | 17.69 | 13.242 | 132.32 | 183.92 | 0.926 | **<0.0001** | 0.6 ± 0.1 |
|  |  | LMA | 122.12 | -1.90 | 12.656 | 101.27 | 142.96 | 0.919 | **<0.0001** | 0.4 ± 0.1 |
| POM | δ^13^C | HMA | 1.78 | -18.07 | 2.804 | 0.41 | 3.14 | 0.329 | **0.015** | 1.0 ± 0.6 |
|  |  | LMA | 74.01 | -8.62 | 3.42 | 27.26 | 120.76 | 0.433 | **0.005** | 0.03 ± 0.02 |
|  | δ^15^N | HMA | 13.46 | 10.60 | 3.699 | 5.60 | 21.32 | 0.475 | **0.003** | 1.1 ± 0.6 |
|  |  | LMA | 279.24 | 33.96 | 3.543 | 108.97 | 449.51 | 0.452 | **0.004** | 0.07 ± 0.03 |

**Supplementary Table S3**

Results of individual two-factor Permutational multivariate analysis of variance (PERMANOVAs) testing for differences in levels of DOM- or POM-derived ^13^C- and ^15^N-enrichment between treatment timepoints (T_0_, T_0.25_, T_0.5_, T_3_, T_48_) and each Region of Interest (ROI) in the sponges *Plakortis angulospiculatus* (HMA) and *Halisarca caerulea* (LMA). d.f. = degrees of freedom, SS = sum of squares, MS = mean sum of squares, *P*_(perm)_ = permutational *P* value, UP = unique permutations. Values in bold are statistically significant (*P*_(perm)_ < 0.05).

|  | Tracer | Element | Source | df | SS | MS | Pseudo-*F* | *P*_(perm)_ | UP |
| --- | --- | --- | --- | --- | --- | --- | --- | --- | --- |
| HMA | DOM | ^13^C | Time | 4 | 1.022 | 0.25549 | 15.753 | **0.0001** | 9944 |
|  |  |  | ROI | 3 | 1.2563 | 0.41875 | 25.819 | **0.0001** | 9952 |
|  |  |  | Ti x ROI | 12 | 2.4776 | 0.20647 | 12.73 | **0.0001** | 9924 |
|  |  |  | Res | 4972 | 80.478 | 0.016219 |  |  |  |
|  |  |  | Total | 4991 | 107.25 |  |  |  |  |
| HMA | DOM | ^15^N | Time | 4 | 4.9939 | 1.2485 | 12.39 | **0.0001** | 9947 |
|  |  |  | ROI | 3 | 9.3143 | 3.1048 | 30.187 | **0.0001** | 9955 |
|  |  |  | Ti x ROI | 12 | 19.263 | 1.6052 | 15.607 | **0.0001** | 9918 |
|  |  |  | Res | 4972 | 510.35 | 0.10285 |  |  |  |
|  |  |  | Total | 4991 | 737.58 |  |  |  |  |
| LMA | DOM | ^13^C | Time | 4 | 2.5997 | 0.64992 | 19.24 | **0.0001** | 9958 |
|  |  |  | ROI | 4 | 4.8463 | 1.2116 | 35.867 | **0.0001** | 9939 |
|  |  |  | Ti x ROI | 16 | 4.1908 | 0.26192 | 7.7538 | **0.0001** | 9916 |
|  |  |  | Res | 1914 | 64.654 | 0.03378 |  |  |  |
|  |  |  | Total | 1938 | 97.432 |  |  |  |  |
| LMA | DOM | ^15^N | Time | 4 | 6.2677 | 1.5669 | 17.708 | **0.0001** | 9954 |
|  |  |  | ROI | 4 | 29.577 | 7.3942 | 83.56 | **0.0001** | 9954 |
|  |  |  | Ti x ROI | 16 | 25.169 | 1.573 | 17.777 | **0.0001** | 9907 |
|  |  |  | Res | 1914 | 169.37 | 0.088489 |  |  |  |
|  |  |  | Total | 1938 | 303.95 |  |  |  |  |
| HMA | POM | ^13^C | Time | 4 | 0.11857 | 0.029643 | 27.314 | **0.001** | 9936 |
|  |  |  | ROI | 3 | 0.011655 | 0.003885 | 3.5798 | **0.03** | 9934 |
|  |  |  | Ti x ROI | 12 | 0.062123 | 0.005177 | 4.7702 | **0.0068** | 9936 |
|  |  |  | Res | 6344 | 6.8447 | 0.001085 |  |  |  |
|  |  |  | Total | 6363 | 8.5916 |  |  |  |  |
| HMA | POM | ^15^N | Time | 4 | 0.30304 | 0.075761 | 10.255 | **0.0186** | 9838 |
|  |  |  | ROI | 3 | 0.95024 | 0.31675 | 42.877 | **0.0002** | 9910 |
|  |  |  | Ti x ROI | 12 | 0.63206 | 0.052672 | 7.1299 | **0.026** | 9902 |
|  |  |  | Res | 6344 | 46.592 | 0.007387 |  |  |  |
|  |  |  | Total | 6363 | 50.761 |  |  |  |  |
| LMA | POM | ^13^C | Time | 4 | 0.60889 | 0.15222 | 6.3419 | **0.0119** | 9933 |
|  |  |  | ROI | 4 | 1.8052 | 0.45131 | 18.803 | **0.0004** | 9943 |
|  |  |  | Ti x ROI | 16 | 1.131 | 0.070688 | 2.945 | **0.0152** | 9937 |
|  |  |  | Res | 1659 | 39.82 | 0.024003 |  |  |  |
|  |  |  | Total | 1683 | 46.386 |  |  |  |  |
| LMA | POM | ^15^N | Time | 4 | 13.114 | 3.2785 | 10.159 | **0.0057** | 9947 |
|  |  |  | ROI | 4 | 18.952 | 4.738 | 14.682 | **0.004** | 9954 |
|  |  |  | Ti x ROI | 16 | 11.372 | 0.71074 | 2.2024 | **0.0383** | 9934 |
|  |  |  | Res | 1659 | 535.37 | 0.32271 |  |  |  |
|  |  |  | Total | 1683 | 620.37 |  |  |  |  |

Analysis performed in Primer V7 [1] with the add on PERMANOVA+ [2]. Resemblance matrices were constructed using Euclidean distances and data were analyzed under the reduced model (9999 permutations) using Type III sum of squares.

**Supplementary Table S4**

Results of *post hoc* pairwise comparisons testing for differences in levels of DOM- or POM-derived ^13^C- and ^15^N-enrichment between treatment timepoints (T_0_, T_0.25_, T_0.5_, T_3_, T_48_) for each Region of Interest (ROI) in the sponges *Plakortis angulospiculatus* (HMA) and *Halisarca caerulea* (LMA). The test statistic is pseudo-*t* (t). Values in bold are statistically significant (*P*_(perm)_ < 0.05).

|  | | ROI | | | | | | |
| --- | --- | --- | --- | --- | --- | --- | --- | --- |
|  |  |  | Choanocyte | | Mesohyl cell | | Microbial symbiont | |
|  |  | Groups | t | *P*_(perm)_ | t | *P*_(perm)_ | t | *P*_(perm)_ |
| HMA | ­DOC | 0, 0.25 | 3.35 | **0.0001** | 1.756 | 0.0823 | 12.57 | **0.0001** |
|  |  | 0, 0.5 | 2.96 | **0.0001** | 1.274 | 0.1981 | 0.383 | 0.7069 |
|  |  | 0, 3 | 5.49 | **0.0001** | 6.088 | **0.0001** | 26.3 | **0.0001** |
|  |  | 0, 48 | 5.12 | **0.0001** | 5.037 | **0.0002** | 25.06 | **0.0001** |
|  |  | 0.25, 0.5 | 1.05 | 0.32 | 1.064 | 0.3179 | 4.49 | **0.0001** |
|  |  | 0.25, 3 | 3.17 | **0.0009** | 6.855 | **0.0001** | 25.87 | **0.0001** |
|  |  | 0.25, 48 | 0.58 | 0.5827 | 3.063 | **0.0042** | 25.37 | **0.0001** |
|  |  | 0.5, 3 | 3.22 | **0.0001** | 2.086 | 0.531 | 16.76 | **0.0001** |
|  |  | 0.5, 48 | 0.74 | 0.4749 | 0.546 | 0.5971 | 16.43 | **0.0001** |
|  |  | 3, 48 | 3.17 | **0.0001** | 9.077 | **0.0001** | 4.337 | **0.0001** |
|  | DON | 0, 0.25 | 3.66 | **0.0001** | 8.214 | **0.0001** | 14.28 | **0.0001** |
|  |  | 0, 0.5 | 3.16 | **0.0001** | 3.08 | **0.0035** | 19.7 | **0.0001** |
|  |  | 0, 3 | 5.37 | **0.0001** | 6.588 | **0.0001** | 25.1 | **0.0001** |
|  |  | 0, 48 | 6.11 | **0.0001** | 0.471 | 0.4187 | 32.7 | **0.0001** |
|  |  | 0.25, 0.5 | 0.84 | 0.4099 | 25.55 | **0.0001** | 8.536 | **0.0001** |
|  |  | 0.25, 3 | 3.46 | **0.0003** | 12.48 | **0.0001** | 24.49 | **0.0001** |
|  |  | 0.25, 48 | 0.21 | 0.8374 | 3.406 | **0.0001** | 33 | **0.0001** |
|  |  | 0.5, 3 | 3.09 | **0.0002** | 5.477 | **0.0001** | 13.43 | **0.0001** |
|  |  | 0.5, 48 | 0.82 | 0.4303 | 0.65 | 0.9898 | 18.96 | **0.0001** |
|  |  | 3, 48 | 3.04 | **0.0001** | 3.407 | **0.0146** | 6.517 | **0.0001** |
|  | POC | 0, 0.25 | 7.6 | **0.0001** | 2.334 | **0.0241** | 4.838 | **0.0001** |
|  |  | 0, 0.5 | 2.8 | **0.0001** | 10.61 | **0.0001** | 19.21 | **0.0001** |
|  |  | 0, 3 | 0.56 | 0.5923 | 2.379 | **0.023** | 20.33 | **0.0001** |
|  |  | 0, 48 | 3.6 | **0.0001** | 0.482 | 0.6381 | 6.177 | **0.0001** |
|  |  | 0.25, 0.5 | 3.06 | **0.0001** | 4.809 | **0.0003** | 15.82 | **0.0001** |
|  |  | 0.25, 3 | 1.38 | 0.1816 | 3.789 | **0.0004** | 28.91 | **0.0001** |
|  |  | 0.25, 48 | 3.76 | **0.0002** | 2.424 | **0.0193** | 11.08 | **0.0001** |
|  |  | 0.5, 3 | 2.93 | **0.0012** | 8.126 | **0.0001** | 31.19 | **0.0001** |
|  |  | 0.5, 48 | 0.37 | 0.7964 | 15 | **0.0001** | 22.05 | **0.0001** |
|  |  | 3, 48 | 2.09 | **0.0332** | 2.99 | **0.0026** | 15.11 | **0.0001** |
|  | PON | 0, 0.25 | 2.48 | **0.0106** | 4.622 | **0.0001** | 2.578 | **0.0105** |
|  |  | 0, 0.5 | 1.11 | 0.1221 | 5.518 | **0.0001** | 5.679 | **0.0001** |
|  |  | 0, 3 | 1.76 | 0.0561 | 3.278 | **0.011** | 1.913 | 0.0563 |
|  |  | 0, 48 | 4.11 | **0.0001** | 9.142 | **0.0001** | 29.6 | **0.0001** |
|  |  | 0.25, 0.5 | 1.47 | **0.0001** | 1.008 | 0.3167 | 3.667 | **0.0003** |
|  |  | 0.25, 3 | 2.34 | **0.0005** | 7.599 | **0.0001** | 0.601 | 0.551 |
|  |  | 0.25, 48 | 5.52 | **0.0001** | 8.176 | **0.0001** | 36.64 | **0.0001** |
|  |  | 0.5, 3 | 0.17 | 0.8905 | 8.516 | **0.0001** | 3.936 | **0.0002** |
|  |  | 0.5, 48 | 1.02 | 0.3403 | 8.631 | **0.0001** | 29.24 | **0.0001** |
|  |  | 3, 48 | 1.15 | 0.2664 | 9.929 | **0.0001** | 32.03 | **0.0001** |
| LMA | DOC | 0, 0.25 | 0.46 | 0.6518 | 1.01 | 0.3174 | 5.92 | **0.0001** |
|  |  | 0, 0.5 | 4.41 | **0.0001** | 7.06 | **0.0001** | 15.93 | **0.0001** |
|  |  | 0, 3 | 10.19 | **0.0001** | 9.03 | **0.0001** | 11.19 | **0.0001** |
|  |  | 0, 48 | 14.41 | **0.0001** | 13.32 | **0.0001** | 15.26 | **0.0001** |
|  |  | 0.25, 0.5 | 7.48 | **0.0001** | 4.71 | **0.0001** | 13.39 | **0.0001** |
|  |  | 0.25, 3 | 17.15 | **0.0001** | 6.19 | **0.0001** | 9.32 | **0.0001** |
|  |  | 0.25, 48 | 24.18 | **0.0001** | 9.25 | **0.0001** | 13.35 | **0.0001** |
|  |  | 0.5, 3 | 0.79 | 0.4633 | 1.06 | 0.3044 | 0.49 | 0.6329 |
|  |  | 0.5, 48 | 1.02 | 0.3309 | 3.53 | **0.0012** | 4.22 | **0.0009** |
|  |  | 3, 48 | 0.01 | 0.9926 | 3.26 | **0.0014** | 4.77 | **0.0001** |
|  | DON | 0, 0.25 | 0.76 | 0.4864 | 1.34 | 0.1933 | 10.16 | **0.0001** |
|  |  | 0, 0.5 | 3.99 | **0.0001** | 3.25 | **0.0001** | 19.61 | **0.0001** |
|  |  | 0, 3 | 8.39 | **0.0001** | 17.88 | **0.0001** | 12.38 | **0.0001** |
|  |  | 0, 48 | 13.22 | **0.0001** | 7.15 | **0.0001** | 19.40 | **0.0001** |
|  |  | 0.25, 0.5 | 6.85 | **0.0001** | 2.36 | **0.0001** | 15.13 | **0.0001** |
|  |  | 0.25, 3 | 14.36 | **0.0001** | 13.31 | **0.0001** | 8.76 | **0.0001** |
|  |  | 0.25, 48 | 22.61 | **0.0001** | 5.15 | **0.0001** | 16.15 | **0.0001** |
|  |  | 0.5, 3 | 3.39 | **0.0006** | 0.73 | 0.4958 | 4.54 | **0.0001** |
|  |  | 0.5, 48 | 3.84 | **0.0003** | 2.19 | **0.0262** | 3.01 | **0.0035** |
|  |  | 3, 48 | 1.00 | 0.323 | 4.65 | **0.0001** | 8.56 | **0.0001** |
|  | POC | 0, 0.25 | 6.56 | **0.0001** | 20.82 | **0.0001** | 9.07 | **0.0001** |
|  |  | 0, 0.5 | 4.86 | **0.0001** | 24.32 | **0.0001** | 9.67 | **0.0001** |
|  |  | 0, 3 | 5.80 | **0.0001** | 15.53 | **0.0001** | 14.07 | **0.0001** |
|  |  | 0, 48 | 17.76 | **0.0001** | 7.84 | **0.0001** | 13.49 | **0.0001** |
|  |  | 0.25, 0.5 | 2.41 | **0.014** | 0.36 | 0.7235 | 0.77 | 0.4452 |
|  |  | 0.25, 3 | 1.90 | 0.0596 | 2.31 | **0.0064** | 5.14 | **0.0001** |
|  |  | 0.25, 48 | 2.29 | **0.0199** | 4.84 | **0.0001** | 6.54 | **0.0001** |
|  |  | 0.5, 3 | 3.96 | **0.0001** | 2.81 | **0.0001** | 7.25 | **0.0001** |
|  |  | 0.5, 48 | 6.45 | **0.001** | 5.36 | **0.0001** | 8.75 | **0.0001** |
|  |  | 3, 48 | 0.85 | 0.3935 | 4.85 | **0.0001** | 3.13 | **0.0013** |
|  | PON | 0, 0.25 | 4.76 | **0.0001** | 7.46 | **0.0001** | 9.54 | **0.0001** |
|  |  | 0, 0.5 | 2.52 | **0.0024** | 3.57 | **0.0002** | 9.52 | **0.0001** |
|  |  | 0, 3 | 5.00 | **0.0001** | 3.67 | **0.0001** | 17.86 | **0.0001** |
|  |  | 0, 48 | 17.96 | **0.0001** | 8.01 | **0.0001** | 27.01 | **0.0001** |
|  |  | 0.25, 0.5 | 2.27 | **0.0188** | 2.71 | **0.0082** | 0.35 | 0.7278 |
|  |  | 0.25, 3 | 1.53 | 0.133 | 2.64 | **0.0001** | 10.74 | **0.0001** |
|  |  | 0.25, 48 | 2.43 | **0.013** | 7.79 | **0.0001** | 18.67 | **0.0001** |
|  |  | 0.5, 3 | 3.72 | **0.0001** | 3.42 | **0.0001** | 13.39 | **0.0001** |
|  |  | 0.5, 48 | 6.54 | **0.0001** | 8.66 | **0.0001** | 24.05 | **0.0001** |

**References**

1. Clarke KR, Gorley RN. PRIMER v7: User Manual/Tutorial. Plymouth, UK. 2015. p. 1–296.

2. Anderson MJ, Gorley RN, Clarke KR. PERMANOVA+ for PRIMER: Guide to software and statistical methods. Plymouth, UK. 2008. p. 1–214.
